# Hotspots of Arctic and sub-Arctic marine sediment organic carbon are dominated by the Baltic, Barents and Chukchi Seas

**DOI:** 10.1038/s43247-026-03720-8

**Published:** 2026-06-19

**Authors:** B. Langley, H. L. Burdett, K. Cameron, T. Juul-Pedersen, A. Rouillard, C. Slaymark, N. A. Kamenos

**Affiliations:** 1 School of Geographical and Earth Sciences, University of Glasgow, Glasgow, Scotland; 2 Umeå Marine Sciences Centre, Umeå University, Norrbyn, Sweden; 3 Department of Ecology, Environment and Geoscience, Umeå University, Umeå, Sweden; 4 Greenland Climate Research Centre, Greenland Institute of Natural Resources, Nuuk, Greenland

**Keywords:** Marine chemistry, Marine chemistry, Carbon cycle

## Abstract

The Arctic and sub-Arctic are warming at least three times faster than the global average, altering terrestrial carbon delivery to the oceans and marine carbon cycling. The sequestration of such carbon into marine sediments is a key contributor to climate regulation. Despite this, the location of organic carbon hotspots at high latitudes are poorly understood, hindering our ability to identify their sensitivity to environmental change. Using quantile regression forests with >13,000 sediment samples, we identify the Baltic, Barents and Chukchi Seas as the dominant high-latitude marine organic carbon hotspots, playing a disproportionately large role in the area-normalised global sedimentary carbon stock. Organic carbon accumulation rates are elevated across shallow shelf environments and coastlines, particularly in proximity to Arctic rivers. Development of organic carbon hotspots reflects both local and external processes, including salinity, mixed layer depth, primary production and sedimentation, demonstrating the importance of coupled land-ocean processes. Uncertainty in future changes to organic carbon cycling and transit pathways, including river transport, is therefore an emerging risk factor for the stability of marine sediment carbon stores. Compounding this, only 10.19% of the surface sediment organic carbon stock is currently within marine protected areas, placing >17 Pg at higher risk of anthropogenic disturbance.

## Abstract (web summary)

The Baltic, Barents, and Chukchi Seas play a significant role in the area-normalised global sedimentary carbon stock, dominating high latitudes, according to analysis of quantile regression forests using sediment samples

## Introduction

Marine sediments serve as a vast sink of organic carbon (OC), receiving inputs from the biological carbon pump, riverine sources, coastal erosion and glacial discharge^1,2^. Following deposition at the seafloor, a proportion of OC is retained within sediments (OC accumulation). A smaller fraction of this accumulated OC is subsequently transferred to deeper sediment horizons, where it can be preserved over millennial timescales (OC burial) representing long-term storage below depths at which OC concentrations remain relatively constant^3,4^. Together, it is estimated that the global OC stock in marine sediments is 2322 Pg OC (upper 1 m)^5^ which is comparable to the terrestrial sediment OC stock (2400 Pg OC^6^) and thus the marine environment plays an important role in the global carbon cycle, including regulating climatic changes^7^. Critically, OC storage in the Arctic and sub-Arctic is of particular importance as higher latitudes are warming at least three times faster than the global average^8^, with direct and indirect consequences for both contemporary OC burial, but also the preservation of previously buried OC^9^. While we have a broad understanding of major pathways, sources and processes contributing to marine OC accumulation at high latitudes^10–12^, the large-scale spatial distribution of sedimentary OC remains poorly constrained, hindering identification of OC hotspots. This limits our ability to assess the sensitivity of drivers generating the OC hotspots, and thus ultimately determine the stability of those stocks in a changing world^9,13^.

Compounding our poor understanding of high-latitude carbon distribution, climate warming is resulting in increased precipitation, terrestrial productivity, glacial and river discharge, permafrost thawing, ocean temperatures, ocean freshening and sea ice decline^8^. Such changes are causing shifts in the delivery of terrestrial OC to the ocean^14^, altered rates of marine primary production^15^ and changing relationships to nutrient, light, oxygen and redox conditions that underpin ecosystem dynamics^16,17^. Projections suggest that the combined terrestrial and marine high latitude carbon sink will reduce under future warming due to coastal erosion, riverine OC outgassing, shifting in phytoplankton community structure and increased OC remineralisation^18,19^.

In parallel with these climate-driven pressures, direct anthropogenic disturbance of marine sediments represents an additional and potentially compounding threat to Arctic and sub-Arctic OC storage. While still debated^20^, there is some evidence that human activities including trawling, mineral extraction and energy installations can disturb sediment OC, leaving it vulnerable to remineralisation and release from the ocean floor^21–23^. Such activities are already widespread in the sub-Arctic (e.g., North Sea^24^) and are projected to increase in the Arctic as a result of diminishing sea ice and a warmer Arctic Ocean^25^. Taken together, Arctic and sub-Arctic sedimentary OC reservoirs will increasingly depend on both the trajectory of climate change and the management of human activities on the seafloor.

Spatial modelling provides a powerful framework for predicting sedimentary OC distribution and has advanced global-scale assessments; however, Arctic regions are typically underrepresented or poorly resolved in such models^5,26^. Previous syntheses of pan-Arctic OC distribution have largely relied on spatial interpolation of surface sediment measurements, such as Bayesian kriging^11^. While useful, these approaches depend primarily on spatial autocorrelation and are limited in their ability to incorporate environmental covariates or capture non-linear relationships. More recently, machine-learning approaches have shown promise in regional-scale assessments of surface sediment OC across the Arctic and sub-Arctic^27–33^. These models can accommodate numerous predictors, represent complex non-linear and interaction effects, and provide metrics of variable importance to identify key predictors of OC distribution. However, to date their application remains largely restricted to regional domains, constraining our ability to determine pan-Arctic and sub-Arctic hotspots and spatial heterogeneity of OC in marine surface sediments.

Our understanding of sedimentary OC distribution beyond surface sediments is even more limited. Most existing datasets and spatial predictions are concentrated within the upper centimetres of sediment^11,27–33^. Although, by necessity, OC stocks have been estimated to depths of up to 1 m in some studies^10^, these assessments often assume vertically uniform OC and do not explicitly account for within sediment variability (downcore) arising from changes in OC sources, depositional environments and post-depositional remineralisation. As a result, OC stored in sub-surface sediments remains poorly characterised.

Here, using 13,662 surface sediment samples (top 10 cm) and 620 sediment cores (up to 1 m) we determine, (1) the location and magnitude of Arctic and sub-Arctic OC hotspots and coldspots, (2) whether surface hotspots persist in sub-surface sediments and (3) the bio-environmental variables underpinning the spatial occurrence of those hotspots and coldspots, resolving their role in global sediment OC dynamics. Using this geospatial context, we assess potential resilience and identify priority regions for consideration in future carbon management strategies.

## Results and discussion

### Organic carbon hotspots in the Arctic and sub-Arctic

#### Organic carbon concentration hotspots

**Fig. 1:**
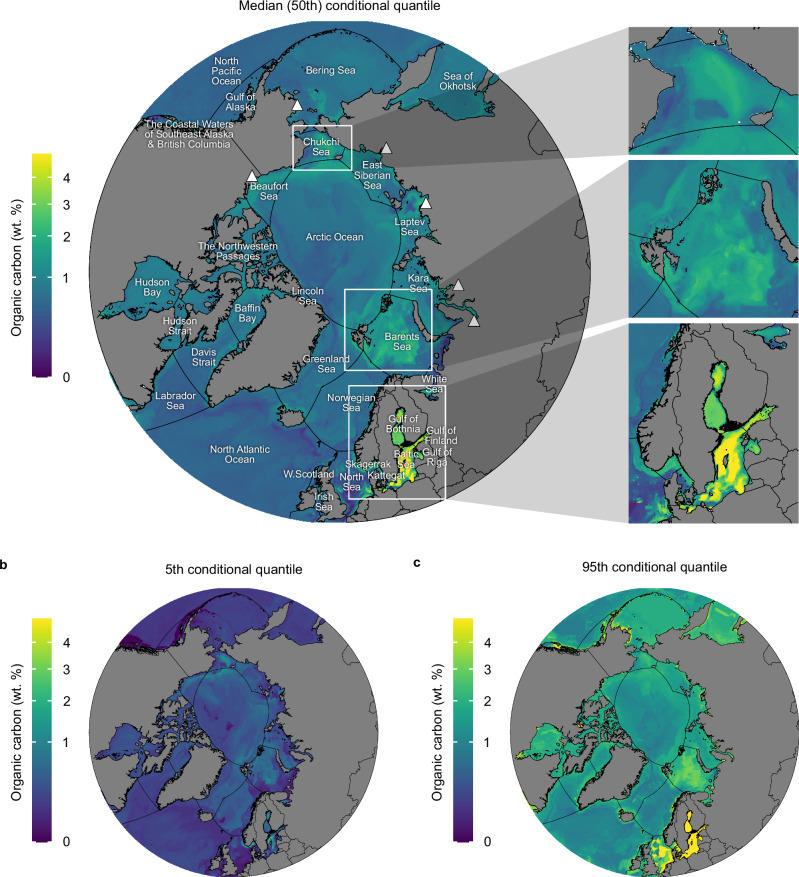
Predicted organic carbon (OC) concentrations in surface sediments. **a** Median (50th conditional quantile) OC concentration in the upper 10 cm of sediment predicted using quantile regression forest (QRF) modelling. Seas are delineated by black lines and labelled in white, and major Arctic rivers are indicated by white triangles. Insets highlight regions of elevated OC concentrations. **b** Lower (5th conditional quantile) and **c** upper (95th conditional quantile) prediction bounds, illustrating the model-based prediction uncertainty in OC concentrations. Note the non-linear colour scale across all panels.

Predicted OC (wt.%) in the upper 10 cm of sediment was spatially heterogeneous across the Arctic and sub-Arctic, ranging between 0 and 23.88 wt.% (Fig. 1a). OC ranged between 0 and 6.48 wt.% at the 5th conditional quantile (Fig. 1b) and 0.06–34.51 wt.% (Fig. 1c) at the 95th quantile. The difference between the 5th and 95th quantiles (i.e., Q95–Q5), representing model-based predictive uncertainty in OC%, spanned 0.03–34.46 wt.%, with largest predictive uncertainty in the Baltic region and North Sea (Fig. S1a).

**Fig. 2:**
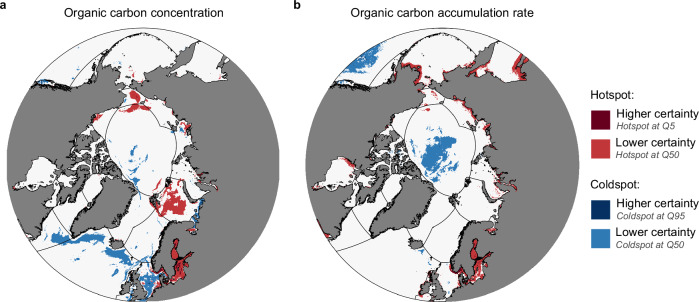
Hotspots and coldspots of organic carbon (OC) in surface sediments. **a** Spatial distribution of hotspots and coldspots of OC concentration in the upper 10 cm of sediment. **b** Spatial distribution of hotspots and coldspots of OC accumulation rate. Hotspots (>95th percentile of median prediction) and coldspots (<5th percentile of median prediction) are classified based on quantile regression forest outputs. Higher certainty hotspots (dark red) and coldspots (dark blue) are defined using the 5th and 95th conditional quantiles, respectively, while lower certainty classes (light red and light blue) are based on the median prediction only. Seas are delineated by black lines.

We identify that OC% hotspots (OC > upper 5% of median prediction; >1.43 wt.%) generally occurred in coastal regions on continental shelves, with spatially extensive OC% hotspots identified in the Baltic region (Fig. 2a), where mean OC% were highest in the Baltic Sea Proper (median: 3.96 wt.%; 5th–95th conditional quantiles: 0.55–9.93 wt.%), Gulf of Finland (median: 3.15 wt.%; 5th–95th conditional quantiles: 0.43–7.04 wt.%), and Gulf of Bothnia (median: 2.63 wt.%; 5th–95th conditional quantiles: 0.51–6.08 wt.%) (Fig. 1). Notably, several smaller Baltic sub-basins exhibited near-complete hotspot coverage, with the entire sediment surface exceeding the OC% hotspot threshold in the Gulf of Riga, and similarly high proportions in the Gulf of Finland (96%) and Gulf of Bothnia (88%) (Fig. 2a).

At higher latitudes, Arctic shelf seas exhibited regionally elevated OC%, with mean OC% at the median quantile of 1.19 (5th–95th conditional quantiles: 0.36–2.35) wt.% in the Barents Sea and 1.09 (5th–95th conditional quantiles: 0.44–1.80) wt.% in the Chukchi Sea (Fig. 1), with extensive areas where OC% exceeded the hotspot threshold (Fig. 2a). Spatially confined hotspots were identified in nearshore regions influenced by major Arctic river inputs, notably the Mackenzie River draining into the Beaufort Sea and the Lena River discharging into the Laptev Sea (Fig. 2a).

Lowest OC% were identified in deeper waters, with spatially large coldspots (OC <lower 5% of median prediction; <0.41 wt.%) identified in sediments in the North Atlantic and Labrador Sea (Fig. 2a). Although the North Sea contains some of the highest predicted OC%, it also exhibits some of the lowest, with large areas classified as coldspots. Consistent with previous predictions^27^, the Norwegian Trough was identified as a hotspot of OC%, while lower concentrations are present in adjacent regions of the Dogger Bank and Southern Bight, extending into the English Channel.

#### Organic carbon accumulation rate hotspots

**Fig. 3:**
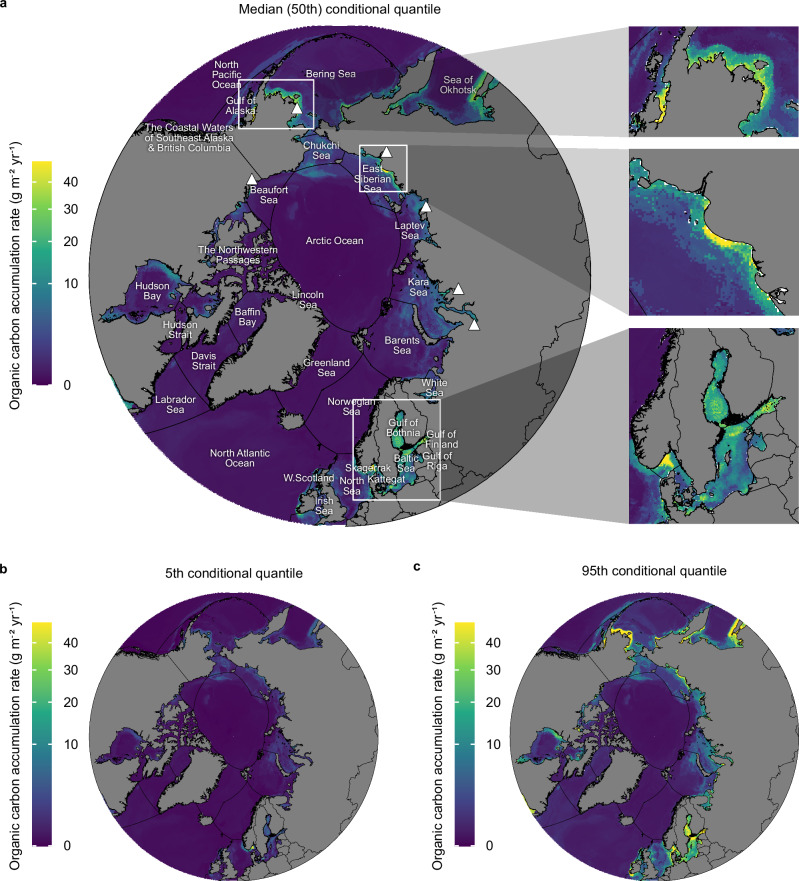
Predicted organic carbon accumulation rate (OCAR) in surface sediments. **a** Median (50th conditional quantile) OCAR from quantile regression forest predicted surface sediment organic carbon concentrations combined with sediment mass accumulation rates^34^. Seas are delineated by black lines and labelled in white, and major Arctic rivers are indicated by white triangles. Insets highlight regions of elevated OCARs. **b** Lower (5th conditional quantile) and **c** upper (95th conditional quantile) prediction bounds, calculated from the corresponding lower and upper bounds of surface sediment OC concentrations. Note the non-linear colour scale across all panels.

Using modelled post-industrial sediment mass accumulation rates (MAR)^34^, alongside our modelled OC%, we generated OCARs that range from 0 g m^−2^ yr^−1^ (North Pacific Ocean and Coastal Waters of Southeast Alaska and British Columbia) to 197 g m^−2^ yr^−1^ (North Sea) (Fig. 3a). OCAR ranged between 0–105 g m^−2^ yr^−1^ at the 5th (Fig. 3b) and 0.0093–1372 g m^−2^ yr^−1^ at the 95th conditional quantile (Fig. 3c). Calculated OCAR values spanned 0.0093–1371 g m^−2^ yr^−1^ within the 5–95th conditional quantiles, with highest prediction uncertainty observed along sub-Arctic coastal regions, including the Bering Sea and Baltic region (Fig. S1b). As uncertainty in modelled MAR^34^ used in our OCAR calculations is greatest in the North Sea, Chukchi Sea, East Siberian Sea and Bering Sea^34^, total OCAR uncertainty in these regions is likely underestimated by the reported prediction intervals.

OCAR hotspots (OCAR > upper 5% of median prediction; >6.07 g m^−2^ yr^−1^) were predominantly concentrated in coastal regions (Fig. 3b), reflecting elevated sediment MARs in these nearshore environments^34^. Highest mean OCARs were predicted for the Skagerrak (median: 26 g m^−2^ yr^−1^; 5th–95th conditional quantiles: 13.82–66.92 g m^−2^ yr^−1^), extending into the Norwegian Trough, with values comparable to those reported in previous mapping studies (19.4 g m^−2^ yr^−1^ across the Norwegian Trough^27^). Elevated OCARs were also observed across Baltic sub-basins, including the Gulf of Finland (median: 22.90 g m^−2^ yr^−1^; 5th–95th conditional quantiles: 3.05–50.95 g m^−2^ yr^−1^) and Gulf of Bothnia (median: 15.90 g m^−2^ yr^−1^; 5^th^–95^th^ conditional quantiles: 3.01–36.73 g m^−2^ yr^−1^). These Baltic regions also exhibited extensive hotspots coverage, with 98% of the Gulf of Finland and 96% of the Gulf of Bothnia exceeding the OCAR hotspot threshold, likely reflecting the combined influence of high OC inputs and rapid accumulation of fine-grained sediments, which together enhance OC accumulation^35^.

#### Organic carbon concentration hotspots in sub-surface sediments

**Fig. 4:**
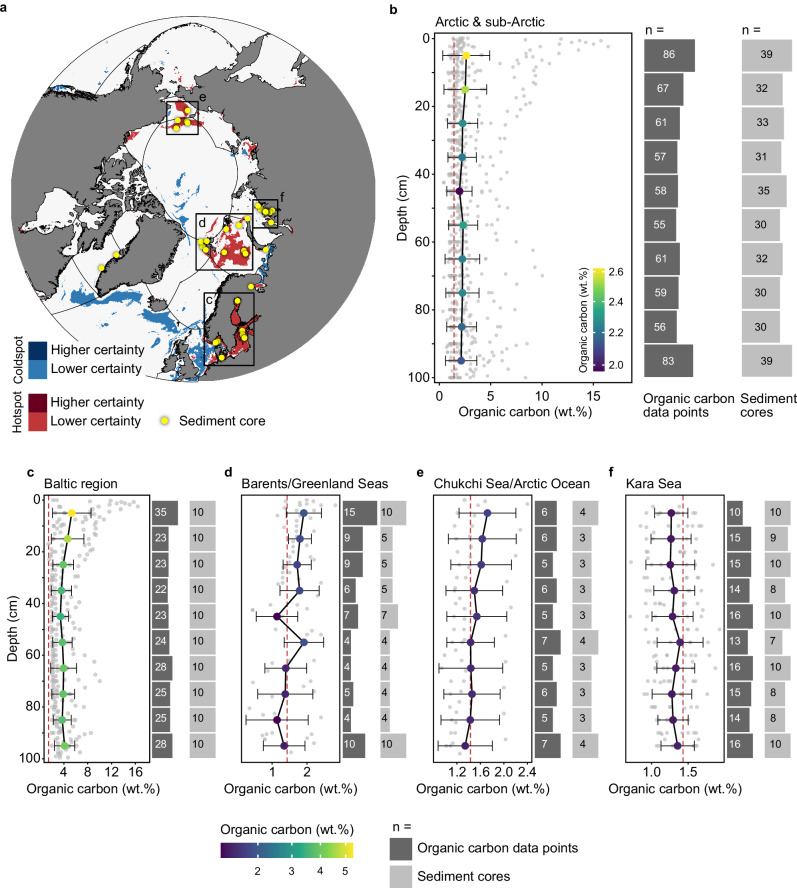
Vertical distribution of organic carbon (OC) under surface sediment OC% hotspots. **a** Map of sediment core locations classified as OC% hotspots. **b–e** Mean OC concentrations in the upper 1 m of sediment, derived from sediment core data. Coloured points indicate mean OC concentration (wt.%) for each 10 cm depth interval with horizontal bars indicating ± 1 standard deviation among cores, grey points represent individual measurements and red dashed line indicates the hotspot threshold defined by the upper 95th percentile of surface sediment OC (OC > 1.43 wt.%). Histogram plots to the right of each profile show the number of OC measurements (dark grey) and cores (light grey) within each depth interval. Panels show **b** Arctic and sub-Arctic, **c** Baltic region, **d** Barents Sea, **e** Chukchi Sea and **f** Kara Sea. Regions are indicated by labelled black boxes in a.

To assess whether sediment surface OC% hotspots reflect persistent sub-surface (and thus temporal) hotspots, we analysed sediment cores from hotspot regions (Fig. 4a). Across hotspot regions, OC% declined with depth, however mean OC% remained above the hotspot threshold (1.43 wt.%) to depths up to 1 m, indicating long-term and sustained OC delivery and burial (Fig. 4b). OC% of cores from the Baltic region, showed a steeper decline with depth (*T*_eff(0–100 cm)_ 77.5 ± 57.0%), while still maintaining concentrations above the hotspot threshold in the upper 1 m (Fig. 4c).

Whilst sediment core data are more limited at higher latitudes, mean OC% across cores from surface hotspots in the Barents and Chukchi Seas fall below the hotspot threshold (wt.%) within the upper 1 m (Fig. 4d, e). This observed decline below the hotspot threshold likely reflects the lower initial (surface at time of deposition) OC concentration, such that modest decreases with sediment depth due to remineralisation may fall below the threshold. Consequently, although transfer efficiencies over the upper 1 m (Barents Sea: *T*_eff(0–100 cm)_ 70.5 ± 36.4%; Chukchi Sea: *T*_eff(0–100 cm)_ 77.9 ± 34.7%) are comparable to that of the Baltic region, OC concentrations fall below the hotspot threshold with depth.

Coastal Arctic sites influenced by large river systems, particularly near the Ob and Yenisey river mouths in the Kara Sea, display near-linear downcore OC profiles with high transfer efficiency (*T*_eff(0–100 cm)_ 106.8 ± 26.1%; Fig. 4f). Values exceeding 100% may reflect higher OC inputs or differences in OC composition at the time of deposition compared to more recently deposited surface sediments, as well as post-depositional processes such as preferential preservation or diagenetic alteration within the sediment column^1,4^. These regions coincide with predicted OCAR hotspots (Fig. 2b), suggesting that rapid sediment accumulation rates contribute to the efficient transfer and preservation of OC along Arctic coasts.

### The wider Arctic and sub-Arctic marine sedimentary organic carbon stock

**Fig. 5:**
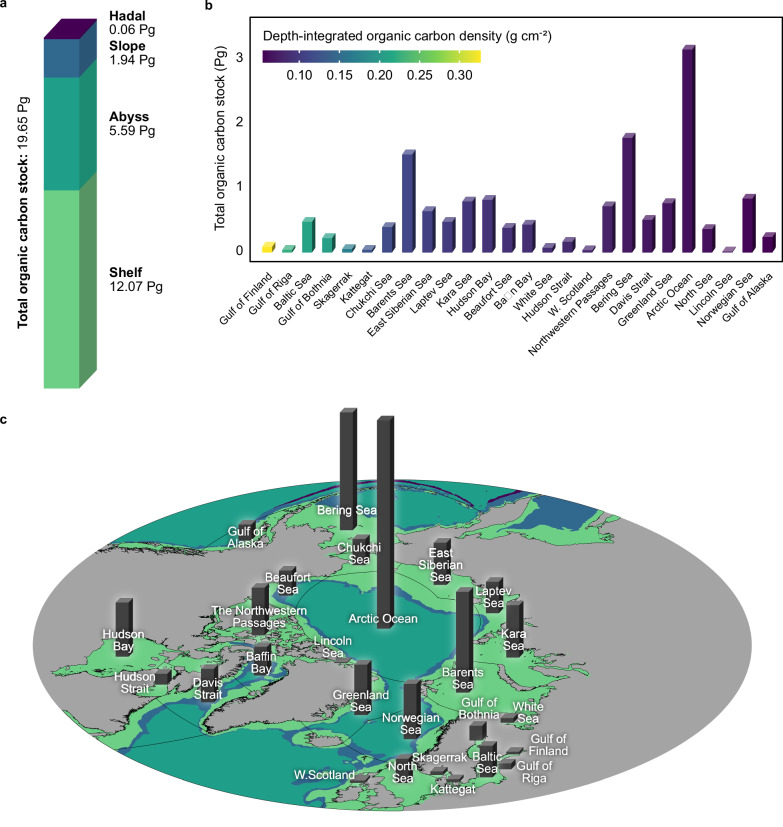
Organic carbon (OC) stock in the upper 10 cm of marine sediments across geomorphic units and seas (50–90°N). **a** Contribution of each geomorphic unit to the total OC stock, expressed as a proportion of the total. **b** Total OC stock within IHO sea areas, ordered and coloured by decreasing mean depth-integrated OC density (g cm^−2^). **c** Spatial distribution of total OC stock by sea, displayed on a flat map of the study region with geomorphic units coloured according to (**a**).

We calculate that 19.65 (5th–95th conditional quantiles: 6.35–45.77) Pg of OC is stored within the top 10 cm of sediments north of 50°N (Fig. 5a; Table S1), representing 13% (5th–95th conditional quantiles: 4–29%) of the global marine sedimentary OC stock (155.8 Pg in the upper 10 cm)^26^. Most of this carbon is stored within the continental shelf (61.41%; median: 12.07 Pg; 5th–95th conditional quantiles: 3.46–29.89 Pg), followed by the abyss (28.45%; median: 5.59 Pg; 5th–95th conditional quantiles: 2.25–11.14 Pg), slope (9.85%; median: 1.94 Pg; 5th–95th conditional quantiles: 0.63–4.60 Pg) and hadal (0.29%; median: 0.06 Pg; 5th–95th conditional quantiles: 0.02–0.14 Pg) areas. As well as the continental shelf representing a large area, it also exhibits the highest depth-integrated OC density (i.e., vertically integrated over the upper 10 cm), storing on average 0.10 (5th–95th conditional quantiles: 0.03–0.24) g cm^−2^ compared to: abyss (median: 0.04 g cm^−2^; 5th–95th conditional quantiles: 0.02–0.09 g cm^−2^), slope (median: 0.06 g cm^−2^; 5th–95th conditional quantiles: 0.02–0.14 g cm^−2^) and hadal (median: 0.04 g cm^−2^; 5th–95th conditional quantiles: 0.01–0.09 g cm^−2^) areas. The elevated OC stock on shelves underscores their importance for high-latitude sedimentary carbon storage. Notably, OC cycling on shelves is highly dynamic given proximity to land, with changes in terrestrial carbon inputs and marine primary production rapidly influencing deposition patterns, as well as frequent and intense human disturbances to the seabed.

Across the Arctic Ocean and its marginal seas (Barents, Kara, Laptev, East Siberian, Chukchi and Beaufort), we estimate a total OC stock of 7.36 (5th–95th conditional quantiles: 2.86–13.49) Pg within the upper 10 cm of sediment (Fig. 5b). More than half of this stock (median: 4.21 Pg; 5th–95th conditional quantiles: 1.39–7.96 Pg) is stored in the marginal seas, despite the greater areal extent of the central Arctic Ocean basin. Although sediment depths differ, our marginal-sea stock estimates show spatial patterns comparable to those reported by Vonk et al. ^10^ for the upper 1 cm and 1 m (e.g., highest in the Barents Sea and lowest in the Beaufort Sea). While Arctic sediments represent 4.72% of the estimated global marine sedimentary OC in the upper 10 cm (global estimates: 155.8 Pg^26^), the sediments are characterised by elevated OC%, with a mean of 0.90 (5th–95th conditional quantiles: 0.36–1.65) wt.% compared to the global average of 0.61 wt.%^26^, reflecting high OC densities per unit area. Thus, Arctic marine sediments play a disproportionately large role in global sedimentary carbon storage relative to their areal extent^3,36^. When extended to adjacent high latitude seas (Baffin Bay, the Bering Sea, Davis Strait, Greenland Sea, Hudson Bay, Hudson Strait, Lincoln Sea, the Norwegian Sea, the Northwest Passages and the White Sea) mean OC concentrations remain elevated (median: 0.83 wt.%; 5th–95th conditional quantiles: 0.30–1.71 wt.%) (Fig. 5b), further reinforcing the broader Arctic-sub-Arctic system as an important region in a global context.

Although the six seas with the highest OC stock per unit area (Gulf of Finland, Gulf of Riga, Baltic Sea, Gulf of Bothnia, Skagerrak and Kattegat) together contain a total sedimentary OC stock of only 0.93 (5th–95th conditional quantiles: 0.16–2.45) Pg, this value is notable given their relatively small combined area (Fig. 5b). Normalised by area, these regions store substantially higher depth-integrated OC densities (median: 0.21 g cm^−2^; 5th–95th conditional quantiles: 0.03–0.55 g cm^−2^) than most other Arctic and sub-Arctic seas.

We considered carbon stocks in the top 10 cm, however, sediment thickness varies across the Arctic and sub-Arctic; most sediments extend well beyond this depth and thus our calculations are likely conservative. Further, we observed that there was general correspondence between the mean OC% integrated over the top 10 cm and the top 1 m of sediment (Fig. S2a), however concentrations of OC% where highest in the top 10 cm of sediments, a pattern particularly evident in OC-rich areas such as the Baltic region (Fig. S2b, c) likely due to OC present below 10 cm sediment depth having undergone further remineralisation^9^. These results suggest that simply extrapolating surface sediment OC concentrations to deeper sediment layers would likely introduce unquantifiable uncertainty in OC stock quantifications.

### Bio-environmental controls on organic carbon hotspot creation

High OC% occurs close to land, with the most pronounced hotspots in the semi-enclosed, shallow Baltic Sea (including Gulf of Finland and Gulf of Bothnia), where restricted exchange with the open ocean and strong freshwater influence generate vertical salinity gradients and water column stratification^37^. These low-salinity, stratified conditions likely reduce oxygen availability at the sediment-water interface, decreasing oxygen penetration depth (OPD) and oxygen exposure time (OET), in turn, suppressing benthic remineralisation and bioturbation and enhancing OC preservation^38^. Consistent with this, surface and benthic salinity ranked the highest-importance predictors of OC% (Fig. S3a) with a non-linear increase in predicted OC% at lower salinities (partial dependence and ICE with random forest; Fig. S3b, c). Although salinity itself is unlikely to exert a direct control on OC accumulation and preservation, it may act as a proxy for stratification and associated redox conditions that regulate sediment OC^37,38^. Furthermore, long histories of human activity in the Baltic catchment, including sustained nutrient loading, have enhanced primary production and OC inputs to sediments, while oxygen depletion reduces OPD and OET, enhancing OC preservation^37^. This contrasts to higher-latitude Arctic shelves where anthropogenic influence is weaker and OC% is generally lower.

OC% hotspots in the Barents and Chukchi Seas (Fig. 2a) may be driven by high inputs of modern, marine-derived organic matter associated with elevated local primary productivity, as indicated by stable carbon (δ^13^C) and radiocarbon (Δ^14^C) signatures from these regions^11,39,40^. Indeed, our model ranked benthic primary production as a key predictor of OC% (Fig. S3e). In the Barents Sea, elevated OC concentrations are concentrated along the southern shelf, in agreement with previous regional assessments^41^, where inflow of relatively warm Atlantic waters limits sea ice cover and sustains high productivity^42^. Similarly, OC% hotspots in the Chukchi Sea align with persistently ice-free or seasonally open-water regions, where enhanced light availability supports high primary production and subsequent OC delivery to the seafloor^43,44^. Mixed layer depth was also an important predictor, with higher OC% concentrations associated with shallower mixed layers, potentially due to the concentration of nutrients and phytoplankton biomass within the euphotic zone, enhancing primary production and the efficiency of OC export to depth^45^. Conversely, in the North Atlantic and Greenland Sea where OC% coldspots occurred (Fig. 2a), deeper mixed layers may promote vertical mixing that redistributes nutrients and biomass over a larger depth range, limiting OC accumulation.

Along the Arctic coastline, OC% and OCAR hotspots occurred close to major Arctic river inputs (Fig. 2). The Mackenzie River and the Lena River contribute the two largest particulate OC fluxes among the six major Arctic rivers, estimated at 758 ± 66 × 10^9 ^g yr^−1^ and 814 ± 52 × 10^9 ^g yr^−1^, respectively^46^, supporting a strong fluvial control on sedimentary OC% deposition and accumulation. Consistent with this interpretation, δ^13^C and Δ^14^C signatures of sediments in these hotspots indicate that much of the OC is terrestrially derived and relatively old^11^, suggesting accumulation of river-mobilised soil and permafrost carbon rather than enhanced in situ marine productivity, in contrast to hotspots in the Barents and Chukchi Seas. While distance to rivers ranked as a mid-importance predictor in our model (Fig. S3a), this likely reflects the limited ability of a single proximity metric to capture localised fluvial influences when predictions are made across a pan-Arctic domain. More broadly, OCAR hotspots along the coast (Fig. 2b) emphasises the role of source-to-sink transit pathways in supplying allochthonous OC to shelf environments^13^. Warming is already enhancing terrestrial OC and sediment fluxes to coastal systems through increased coastal erosion^47–49^ and fluvial^50,51^ and glacial^52,53^ discharge, potentially contributing to elevated OCARs predicted along the Arctic coastline. Given the large carbon stocks stored in northern circumpolar permafrost soils (472 ± 27 Pg of OC to a 1 m soil depth)^54^, continued warming may enhance terrestrial OC fluxes to coastal systems, with implications for the composition and magnitude of OC delivered to Arctic shelf sediments. As these regions are undergoing rapid change, they are also likely associated with greater uncertainty in model estimates.

Marked spatial heterogeneity in OC% is evident within individual seas, most notably in the North Sea, where some of the highest and lowest predicted OC% values occur in close proximity (Fig. 1). This pronounced variability likely arises from differences in water depth and hydrographic conditions that regulate sediment transport, seafloor disturbance and depositional regimes, with decades of demersal fishing disturbance in shallow, heavily trawled areas potentially modulating OC%^23,24,55^. Whilst predictions indicate spatial variability, the available dataset may not fully capture the heterogeneity of depositional environments across basins, potentially introducing unquantifiable biases in model predictions of OC%.

### Organic carbon preservation

Mismatches between hotspots of OC% and OCAR (Fig. 2) indicate that regions of high OC% concentration in surface sediments do not necessarily correspond to high OC input. These differences are primarily driven by variability in sediment MARs, whereby high sedimentation enhances OCAR while diluting OC% through increased mineral input. Sedimentation rate also influences OC preservation by modulating the residence time of OC within the active remineralisation zone, with more rapid sedimentation potentially reducing exposure to degradation. As a result, spatial variability in sedimentation contributes to both dilution and preservation effects, influencing the distribution in surface sediments.

Compiled data across the study region show OC% generally declines below the upper 10 cm across shelf, slope and abyssal environments (Fig. S4). This pattern is consistent with the zone of most active remineralisation being concentrated near the sediment surface, below which OC remains lower but relatively stable due to the preferential loss of labile OC and reduced degradation rates at depth^56^. Shelf sediments exhibit the most rapid decreases in OC% concentration with depth (Fig. S4), reflecting high near-surface OC inputs combined with elevated remineralisation rates and physical disturbance^55,56^. In contrast, below 10 cm, OC% concentrations stabilise in slope and abyssal settings (Fig. S4d, e), suggesting greater long-term preservation under lower-energy and disturbance conditions. Nevertheless, even at sub-surface depths approaching 1 m, shelf sediments retain higher OC concentrations than slope and abyssal environments (Fig. S4c–e), owing to their higher OC inputs and sediment accumulation, underscoring their role as important, albeit dynamic, OC reservoirs.

Sedimentation rates across the Arctic and sub-Arctic range from near-zero values in deep basins to rates of up to 3.97 cm yr^−1^ in shallow coastal regions^57^. Consistent with this, our assessment of radiocarbon ages from modelling indicated that sediment ages at 1 m depth spanned three orders of magnitude, from 11 to 26,000 cal year BP1950 (i.e., 24,050 BCE–1939 CE) (Fig. S5). This means there are substantial differences in the length of time OC has been available for remineralisation, as well as the climatic conditions affecting delivery and burial of OC. The age of sediments found at 1 m depth across all dated cores suggests that deposition occurred at periods ranging from the Last Glacial Maximum (19–23 ka years ago), throughout the Holocene (11.7 ka years ago), and in the relatively warm present interglacial period^58,59^.

Recent ^14^C age modelling of riverine-discharged OC has shown that high Arctic OC delivered by rivers is likely to be older refractory carbon derived from permafrost melt, while younger more reactive carbon may increase in proportion in sub-polar latitudes^60^. Hence, OC may reach marine sediments at an already old and refractory state in river-dominated high Arctic areas, which could partly explain the large range of radiocarbon-derived ages we observed at 1 m sediment depth (Fig. S5). However, it is likely a restricted component of that variability as there is not a consistent spatial pattern with the oldest ages (at 1 m) in the high Arctic and younger ages (at 1 m) in the sub-Arctic (Fig. S5).

Variability in the range of sediment carbon ages within a region suggests the incorporation of both autochthonous and allochthonous OC into sediment deposits. In some settings, such as under macroalgal or seagrass beds, autochthonous OC can be a major carbon source^13^, whereas in high latitude shelf seas, the combination of marine and terrestrial inputs results in a range of OC ages at the same sediment depth^10^. OC age of such stocks thus reflects the integrated effects of burial history, sediment mixing and incorporation of pre-aged material. Understanding both spatial and temporal variations in sedimentary OC distribution and age is therefore essential for informed management strategies to protect carbon stocks.

### The future of Arctic and sub-Arctic marine sedimentary organic carbon stock

The vulnerability of this carbon stock and the future source and quantity of new OC inputs may be altered by climate change^9^. Indeed, our results suggest that climate-sensitive controls are among the strongest predictors of OC% at large spatial scales (Fig. S3a).

The main predictors of OC% in our model (salinity, mixed layer depth, temperature and primary production; Fig. S2) are already undergoing rapid change across the Arctic and sub-Arctic in response to climate change, which may have implications for future sedimentary OC storage. Freshening of the Arctic Ocean and increased freshwater export to the North Atlantic^37,61^ is strengthening upper-ocean stratification and shoaling the mixed layer depth^62^, directly influencing key predictors of OC% in this study. Contemporary conditions in the Baltic Sea, characterised by strong salinity gradients, persistent stratification and associated hypoxia^37^, may partially reflect how Arctic regions may evolve in the future^63^.

Temperature and primary production are also undergoing tightly coupled changes across high-latitudes. Increasing ocean temperatures^8,64^, together with influxes of warmer Atlantic waters (Atlantification)^65^, are altering the heat balance of the Arctic Ocean, particularly in the Barents Sea^66^, making this region a hotspot of both sedimentary OC% and climate warming. These physical changes have downstream biological consequences with primary production increasing due to reduced sea ice extent and a lengthening growing season^44,67^, advances in the onset of phytoplankton blooms^68^ and in some regions, the emergence of secondary blooms^69^. Such shifts in productivity and ecosystem dynamics may influence OC supply to sediments, however the net effect on OC storage is complex with evidence of warming and marine heatwaves enhancing benthic remineralisation and bioturbation, contributing to increased OC degradation^70^. The balance between these processes will determine whether future changes in bio-environmental conditions amplify or weaken the OC hotspots identified here.

For carbon accounting it is important to consider the age of the supplied OC, as this may influence its susceptibility to remineralisation following disturbance. We show (Fig. S5) that areas on the boundary between the sub-Arctic and Arctic more consistently contain younger OC stocks, with potential implications for their lability. Further, modelling studies^60^ suggest that, despite a high burial efficiency, high Arctic river-derived OC may have little impact on reducing recent CO_2_ emissions because of the old age of that delivered carbon. However, the burial of this older carbon may still pay an important role in limiting present and future CO_2_ release, particularly where it has been preserved in frozen reservoirs and remains relatively labile and susceptible to degradation^71^.

Our findings suggest that just 10.19% (median: 2.00 Pg; 5th–95th conditional quantiles: 0.46–6.41 Pg in top 1 m) of the calculated total OC stock across the Arctic and sub-Arctic is currently safeguarded within protected areas defined by the World Database of Protected Areas and World Database on Other Effective Area-based Conservation Measures^72^. The majority of the Arctic and sub-Arctic OC store (17.65 Pg in top 10 cm) therefore remains unprotected from seabed disturbance and recent evidence suggests that even where protection exists, it may only be marginally effective^73^.

While at present sub-Arctic seas are widely impacted by anthropogenic activities^24^, the Arctic remains an area of lower impact. However, such disturbances are projected to increase as a result of diminishing sea ice and a warmer Arctic Ocean^25^. Given that coastal management at lower latitudes has proven effective in protecting carbon stocks^74^, there is a need to consider similar strategies for Arctic and sub-Arctic carbon stocks. Based on our assessment of OC% and OCAR hotspots (Fig. 2), we have identified two areas for primary consideration in this context:

1. The high Arctic continental shelf – a valuable and extensive store of young and old carbon that may be vulnerable to future anthropogenic disturbances.
2. Sub-Arctic hotspots of OC% and OCAR – valuable stores of predominantly young carbon, yet ongoing and frequent human disturbances may diminish their potential to contribute to atmospheric CO_2_ removal.

### Conclusions

Our study provides new insights into the spatial dynamics of OC storage and accumulation, along with their controls in marine environments across high latitudes by combining numerical modelling with empirical sediment OC data. QRF modelling predicts that hotspots of surface and sub-surface sediment OC occur in coastal and shelf seas, where the risks of remineralisation by climate change (e.g., warming) and remobilisation by human disturbance are greatest. Localised environmental conditions, such as pronounced salinity gradients in the Baltic Sea and elevated particle inputs at river mouths strongly influence OC% and OCAR and drive spatial variability across the region, highlighting the complexity of sediment OC dynamics. We calculate that 19.65 (6.35–45.77) Pg of OC is stored within the top 10 cm of sediments north of 50°N, representing 13% (4–29%) of the global marine sedimentary OC stock^26^. Importantly, only 10.19% of this Arctic and sub-Arctic carbon stock is currently in protected areas, underscoring the need for continued discussions as to whether these stocks require protection from projected future disturbance.

## Methods

### Data collection

#### Surface sediment OC

To spatially predict OC in surface sediments, OC concentrations (weight percent, wt.%) from 50 to 90°N were compiled from global and regional sediment databases (Fig. S6a). Data were derived from the CASCADE database (The Circum-Arctic Sediment CArbon DatabasE)^11^, which provides OC measurements for surface sediments across the Arctic Ocean and its marginal seas (Beaufort, Chukchi, East Siberian, Laptev, Kara and Barents Seas, as well as the Canadian Arctic Archipelago). To extend coverage into sub-Arctic regions, data were supplemented with the global MOSAIC v2.0 database (Modern Ocean Sediment Archive and Inventory of Carbon)^75^ and several regional compilations and datasets^27–29,76–78^. Duplicate records where longitude, latitude and OC wt.% were identical were identified and removed. Surface sediments were defined as the upper 10 cm of the sediment column, and where multiple data existed within this interval at a given sampling site, mean OC concentration was calculated.

#### Sediment core OC

To investigate OC in deeper sediments, we constructed a new database of sediment cores (Fig. S6b), as existing sediment core datasets are more limited in spatial extent and coverage than those available for surface sediments. A systematic review of literature in Scopus was conducted (October 2023) and titles and abstracts were screened to identify relevant articles, of which OC% data was extracted. When raw OC data was not provided in publications or as supplementary material, corresponding authors were contacted. A search was performed in PANGAEA (June 2023 and March 2024) for sediment core OC% datasets. Cruise reports from research expeditions of the Ocean Drilling Programme (1985–2003), Integrated Ocean Drilling Program (2003–2013), GEOMAR and JAMSTEC were also consulted for additional cores. OC% data, alongside geochemistry, spatial, methodological and chronological information was extracted and compiled into a database. Additional information on data collection and database construction is available in the supplementary information (S1; S2; Table S2).

#### OC data quality control

Surface sediment and sediment core OC datasets were compiled from multiple sources with differing sampling and analytical approaches. Consequently, variability associated with differences in measurement protocols and data reporting may be present. All data were screened for implausible values through inspection of data distributions (Fig. S6c, d) and cross-checked against associated metadata. No data were excluded solely based on magnitude, as extreme values may reflect genuine environmental variability, particularly given the wide range of depositional environments represented in the dataset.

#### Predictor variables for spatial OC upscaling

Variables used to predict surface sediment OC concentrations included sediment and seafloor properties that affect the accumulation and preservation of OC (bathymetry, slope, porosity, grain-size, sediment accumulation rate and bacteria biomass), water column conditions that influences production, transport and remineralisation (temperature, salinity, oxygen, pH, primary production, chlorophyll, mixed layer depth and current velocity), sea ice conditions that affect productivity and delivery of OC to the seafloor (concentration and thickness) and spatial indicators that capture terrestrial inputs and landscape influence on OC (distance to coasts, rivers, glaciers and permafrost) (Table S3). Mineralogical controls on OC preservation (e.g., via metal oxides and clay minerals^79–82^) are not explicitly represented in the predictor variables used due to limited spatial availability of datasets at the scale of this analysis. Their influence may therefore only be indirectly captured through environmental predictors (e.g., sediment accumulation rate). All predictor variable raster layers were reprojected to a WGS84 Arctic Polar Stereographic (EPSG:3995) coordinate system and resampled to a 10 km grid (based on the native resolution of the coarsest predictor variable dataset) using bilinear interpolation. The resulting layers were cropped to 50 to 90°N and assembled into a raster stack (Fig. S7).

### Surface sediment OC quantile regression forest modelling

OC distributions from 50 to 90°N were predicted using quantile regression forest (QRF) models. Random forests^83^ is a machine learning (ML) algorithm that uses an ensemble of decision trees to predict numerical values. It constructs multiple decision trees trained on subsets of the data (bootstrap sample) and creates an average of their predictions. QRF modelling extends this framework by retaining the full distribution of predictions from individual trees, allowing conditional quantiles (e.g., median, interquartile range) of the response variable to be estimated, rather than only mean predictions^84^. Given that the response variable (OC wt.%) is highly skewed, QRF offers an advantage by estimating quantiles that reflect the asymmetric distribution of values, yielding more reliable central predictions than mean-based random forests.

Other ML algorithms, including a gradient boosting algorithm (XGBoost) and a feedforward neural network, were also evaluated to identify the model with the highest predictive performance. All models were trained using the same predictor variables and training-testing data split and model performance was evaluated using *R*^2^ and root-mean-square error (RMSE) on withheld test data. A comparison of the models showed similar overall performance, however, the random forest model exhibited the highest predictive ability, with the highest *R*^2^ and lowest RMSE (Fig. S8). Consequently, QRF modelling was selected for predicting OC concentrations. The use of random forest-based approaches, including QRF, is well established in spatial predictions of sedimentary and biogeochemical variables, where they have demonstrated strong predictive performance across a range of settings^27,28,30,32,33,85^.

While random forest models are robust to correlated predictor variables, high correlation can affect the interpretation of variable importance therefore predictor variables that were highly correlated were removed. A random sample of 5000 pixels was extracted from the raster layers, and Pearson’s correlation coefficient (*r*) was calculated for all variable pairs. Surface primary production and surface chlorophyll, and distance to coast and distance to permafrost were highly correlated (*r* > 0.95); consequently, surface primary production and distance to permafrost were removed from the model, as the remaining variables were considered to better represent or have a larger influence on OC dynamics in marine sediments.

To tune the model and assess performance, 80% of the data were used for training and 20% were withheld for testing^86^. Hyperparameter tuning was performed on the training data to optimise the minimum number of data points required to split a node (min_n), number of predictor variables considered at each split (mtry) and number of trees (n_trees)^86^. Initially, a broad grid search was conducted, exploring combinations of min_n (1–40), mtry (1–22) and n_trees (500–2000), and models were evaluated based on out-of-bag (OOB) *R*^2^ and RMSE. This process was repeated twice more, each time narrowing the search around the best performing parameters. A final tuning step was performed to determine the optimal number of trees. The selected hyperparameters were min_n = 25, mtry = 9, and n_trees = 2000.

The final QRF model was trained on the full 80% training dataset using these parameters. Model performance was evaluated on the independent 20% hold-out test set, yielding *R*^2^ = 0.58 and RMSE = 0.96 wt.% on the median (50th conditional quantile). Model performance was moderate, but comparable to previous OC% mapping studies using similar random forest-based approaches^28^ and likely reflects the wide range of environmental and depositional conditions across the study area.

OC wt.% was predicted using the 50th quantile (median) of the QRF output. Prediction intervals were derived from the 5th and 95th conditional quantiles, which define lower and upper prediction bounds such that 90% of the conditional response distribution is expected to fall between these values, with 5% of outcomes expected to fall below and above these bounds, respectively. The range between the 5th and 95th quantiles was used to represent model-based predictive uncertainty in OC%. This approach captures variability arising from the model structure and from observed variability in OC in the training data for similar predictor conditions, but does not account for uncertainty in predictor variables, measurement error in OC observations or broader limitations related to spatial sampling bias.

#### Predictor variable importance

To assess the relative contribution of predictor variables to OC distribution, we calculated permutation-based variable importance^83^. In this approach, the values of each predictor are randomly permuted one at a time, and the resulting decrease in model performance is used as a measure of the predictor’s importance. The partial dependence of each predictor variable was evaluated to assess its marginal effect on the predicted OC concentrations. This approach enables the identification of non-linear relationships and the relative influence of each predictor, while holding all other variables constant. Individual Conditional Expectation (ICE) plots were also produced, which display prediction responses for individual observations and allow variability around the average partial dependence to be assessed.

### OC accumulation rates

OCAR (g cm^−2^ yr^−1^) was calculated following Eq. 1 where MAR is gridded sediment mass accumulation rate (g cm^−2^ yr^−1^) from Restreppo et al.^34^ and OC is organic carbon concentration calculated from our QRF model (expressed as a decimal proportion). Calculations were performed using the median (50th quantile) as well as the 5th and 95th quantile predictions of OC to quantify the range of predicted values. This range reflects model-based predictive uncertainty in OC derived from the QRF model and does not incorporate uncertainty in MAR. In addition, this approach does not explicitly account for areas undergoing net erosion, as negative MARs are not resolved in the MAR dataset^34^. OCAR was reported as g m^−2^ yr^−1^ by multiplying by 10,000. Raster layers of OCAR and associated prediction intervals were constructed with a resolution of 10 km, corresponding to a pixel size of 10 km × 10 km.

1 $${{{\rm{OCAR}}}}={{{\rm{MAR}}}}* {{{\rm{OC}}}}$$

### Hotspot and coldspot identification

High and low concentrations of OC can be characterised as “hotspots” and “coldspots”, respectively^30,32^. Following this concept, surface sediment OC hotspots were defined as raster grid cells exceeding the 95th percentile of all predicted pixel values from the median (50th) predicted OC conditional quantile (i.e., the upper 5% of the distribution), with “coldspots” defined as cells below the 5th percentile (i.e., the lower 5% of the distribution). Uncertainty was assessed using conditional quantiles from the QRF model. Hotspots and coldspots were considered higher certainty only where the 5th (hotspots) or 95th (coldspots) conditional quantile lay beyond the respective threshold, and lower certainty where the median conditional quantile met the threshold criterion. Hotspots for OCARs were identified using the same percentile-based thresholding approach.

Hotspot occurrence at the median conditional quantile was summarised for individual seas, as defined by the IHO Sea Areas (v.3) classification^87^, by calculating both the absolute area of hotspot grid cells and the proportion of raster cells classified as hotspots within each sea. The proportional metric was used to normalise for differences in total sea area.

### OC stock

Sediment depth-integrated OC density (g cm^−2^) in the upper 10 cm was estimated by firstly calculating dry bulk density (${{{\rm{\rho }}}}{{{\rm{d}}}}$) using gridded porosity (${{{\rm{\phi }}}}$) from Martin et al.^88^ (expressed as a decimal proportion) and a density of dry solids (${{{\rm{\rho }}}}{{{\rm{s}}}}$) of 2.6 g cm^−3^ (Eq. 2), following methods of Parameswaran et al. Dry bulk density was multiplied by predicted OC (expressed as a decimal proportion) and the depth interval of interest (10 cm) to estimate depth-integrated OC density (g cm^−2^; Eq. 3). Depth-integrated OC density prediction intervals were derived from the 5th and 95th quantile predictions of OC%; these do not account for uncertainty in dry bulk density.

2 $${{{\rm{\rho }}}}{{{\rm{d}}}}=\left(1-{{{\rm{\phi }}}}\right)* {{{\rm{\rho }}}}{{{\rm{s}}}}$$

3 $${{{\rm{OC\; density}}}}={{{\rm{\rho }}}}{{{\rm{d}}}}* {{{\rm{OC}}}}* {{{\rm{depth}}}}$$

Total OC stock (Pg) in the upper 10 cm of sediments across the Arctic and sub-Arctic (50–90°N) was calculated by multiplying OC density (g cm^−2^) by pixel area (10 km × 10 km) and summing across all grid cells. Total OC stocks in geomorphic units (continental shelf, continental slope, abyss and hadal) were calculated using units defined by Harris et al.^89^ and estimated for IHO seas^87^. OC stock in protected areas is estimated using the World Database on Protected Areas (WDPA) and the World Database on Other Effective Area-based Conservation Measures (WD-OECM)^72^.

### Sediment core OC concentrations and transfer efficiency

OC (%) was assessed in the upper 1 m of sediment cores across the study region. Sediment depths were binned into 10 cm intervals to standardise depth horizons across cores with variable sampling resolution. To account for uneven sampling density within cores, OC values were first averaged within each core and depth bin, yielding a single OC value per core per depth interval. Analyses were restricted to cores that contained OC measurements in both the surface (0–10 cm) and deep (90–100 cm) intervals to ensure consistent vertical coverage.

Transfer efficiency (*T*_eff_), defined as the fraction of OC flux transiting a specified sediment depth horizon^90^, was calculated for cores located within OC% hotspots. Due to the limited number of dated cores, *T*_eff_ was examined as a function of depth rather than a temporal *T*_eff_ metric. *T*_eff_ was calculated from the 0–10 cm depth interval to the 90–100 cm depth interval using depth-binned mean OC concentrations averaged across all sediment cores, following Eq. 4. Depth-binned mean OC values were used rather than the mean of individual core specific *T*_eff_ to reduce the sensitivity to outliers. Uncertainty in *T*_eff_ was estimated by propagation of error from the standard deviations of OC% in the 0–10 cm and 90–100 cm depth intervals.

4 $${T}_{{{\mathrm{eff}}}(0-100{{\mathrm{cm}}})}=\frac{{{OC}}_{90-100{{\mathrm{cm}}}}}{{{OC}}_{0-10{{\mathrm{cm}}}}}* 100$$

### Age-modelling

Age-depth models were constructed for cores with available uncalibrated radiocarbon ages. Ages were re-calibrated using the Marine20 curve^80^ in OxCal (v.4.4)^91^, applying a marine reservoir effect (ΔR) using the ΔR in closest proximity to the core from the 14CHRONO Marine20 Reservoir Database^92^. A P_Sequence depositional model was applied, assuming continuous sediment accumulation constrained by stratigraphic depth. Replicate measurements from identical horizons were combined using R_Combine. A basal boundary was defined at the deepest sampled depth of each core, and the upper boundary of the sequence was constrained using a calendar-age prior corresponding to the year of core collection ( ± 1 year). Model resolution was set to 20 years. Mean modelled age in calibrated years Before Present (cal year BP) at 1 m depth was extracted for these cores.

### Software

Random forest modelling and analysis was performed in R statistical software (v.4.5.2) using the following packages; dplyr for data manipulation^93^, terra package for rater data handling^94^, sf for analysis of spatial vector data^95^, ranger for fast implementation of random forests^96^, XGBoost for extreme gradient boosting^97^, nnet for neural networks^98^, vip for variable importance plots^99^, DALEX for partial dependence plots^100^, rsample for resampling^101^, corro assess correlations in the data^102^ and ggplot2 for data visualisation^103^. Raster calculations were performed in ArcGIS Pro (v.3.4.3), using the Raster Calculator (Spatial Analyst).

## Supplementary information

Transparent Peer Review file

Supplementary Information

## Data Availability

The surface sediment and sediment core OC datasets, predictor variable raster layers and R scripts used for QRF modelling are available at Figshare 10.6084/m9.figshare.31126942.
