# Supplementary material for: Hotspots of Arctic and sub-Arctic marine sediment organic carbon are dominated by the Baltic, Barents and Chukchi Seas
Source: Commun Earth Environ. 2026 Jun 19;7(1):529. doi: 10.1038/s43247-026-03720-8 (PMC13278947; doi:10.1038/s43247-026-03720-8)
Supplement: Supplementary file 2 — Supplementary Information [file 43247_2026_3720_MOESM2_ESM.pdf]

SUPPLEMENTARY MATERIAL

Hotspots of Arctic and sub-Arctic marine sediment organic carbon are dominated by the Baltic, Barents and Chukchi Seas

Langley, B.[1], Burdett, H.L. [2,3], Cameron, K.[1], Juul-Pedersen, T.[4], Rouillard, A.[2,3], Slaymark, C.[1], Kamenos, N.A.[2,3*]

Affiliations

[1]School of Geographical and Earth Sciences, University of Glasgow, Glasgow, G12 8QQ, Scotland

[2]Umeå Marine Sciences Centre, Umeå University, SE-905 71, Norrbyn, Sweden

[3]Department of Ecology, Environment and Geoscience, Umeå University, SE-907 36, Umeå, Sweden

[4]Greenland Climate Research Centre, Greenland Institute of Natural Resources, Nuuk, Greenland

Communication

nick.kamenos@umu.se

Supplementary Figures:

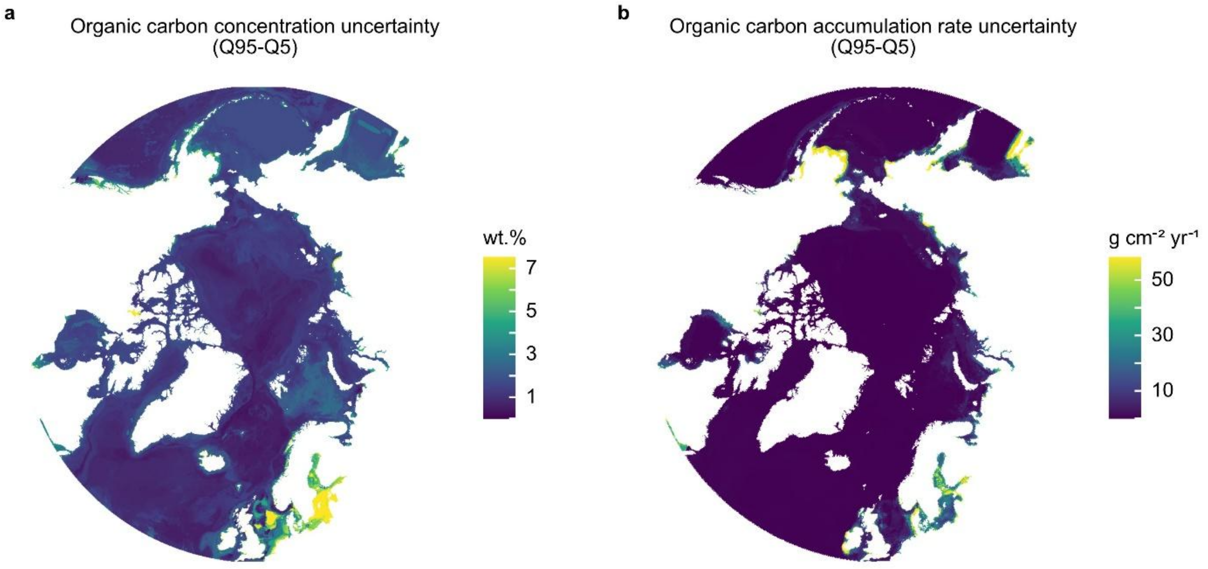

Figure S1: Quantile regression forest model-based predictive uncertainty expressed as the difference between the 95th and 5th conditional quantiles. a) *Organic carbon concentration.* ***b)*** *Organic carbon accumulation rate.*

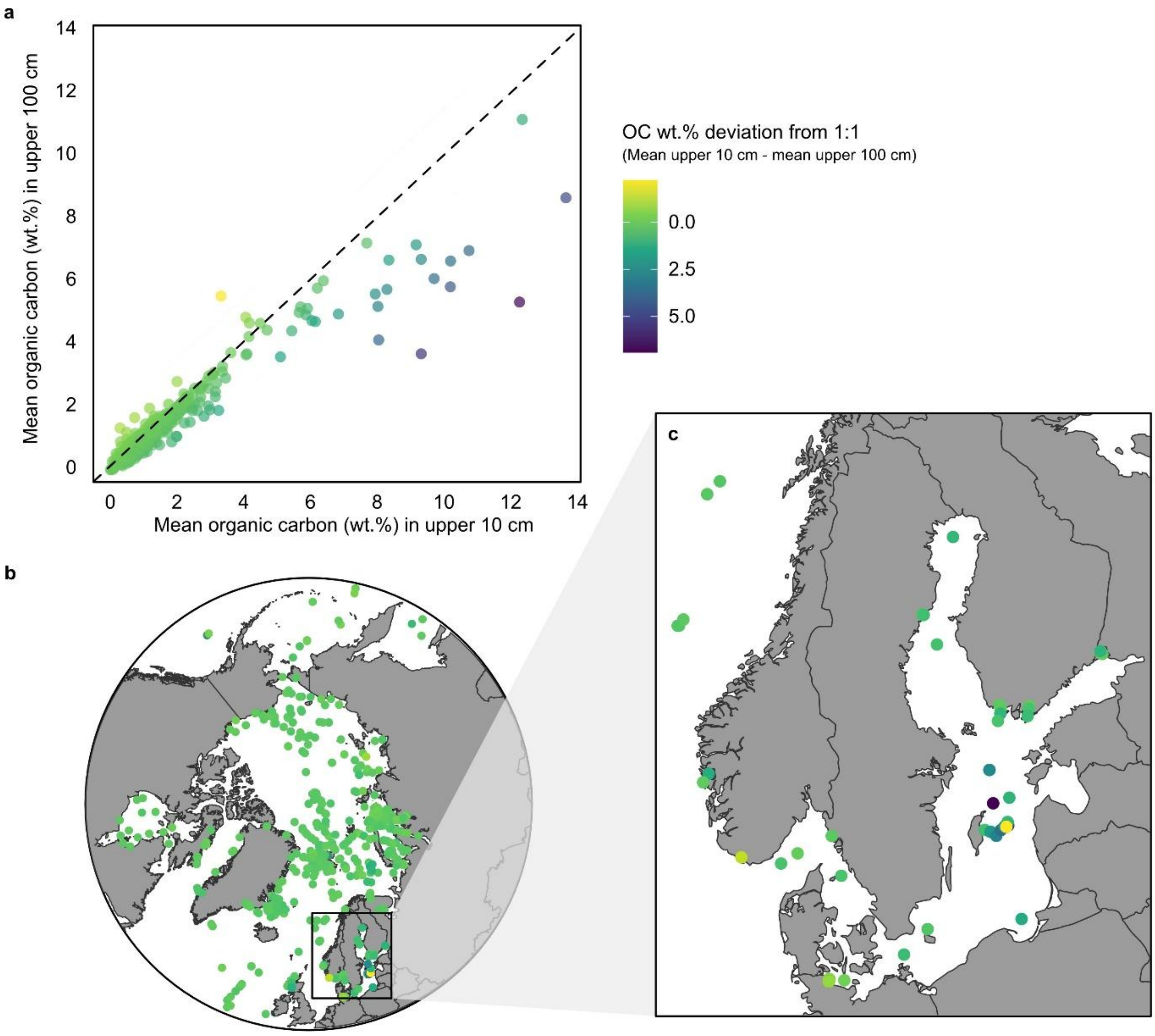

Figure S2: Comparison of organic carbon (OC) in surface and upper 1 m of marine sediments. a) *Relationship between mean OC concentration in the upper 10 cm of sediment and mean OC concentration integrated over the upper 1 m (including the upper 10 cm). The dashed line indicates the 1:1 relationship, where OC concentrations in the upper 10 cm equal those over the full 1 m. Colours denote deviation from the 1:1 line, highlighting differences between surface and deeper sediments and potential uncertainty associated with extrapolating surface OC to depth.* ***b)*** *Spatial distribution of sediment cores coloured by their deviation from the 1:1 line in panel a, with* ***b)*** *highlighting the Baltic Sea region, where the greatest divergence between surface (0–10 cm) and subsurface (0–1 m) OC concentrations is observed.*

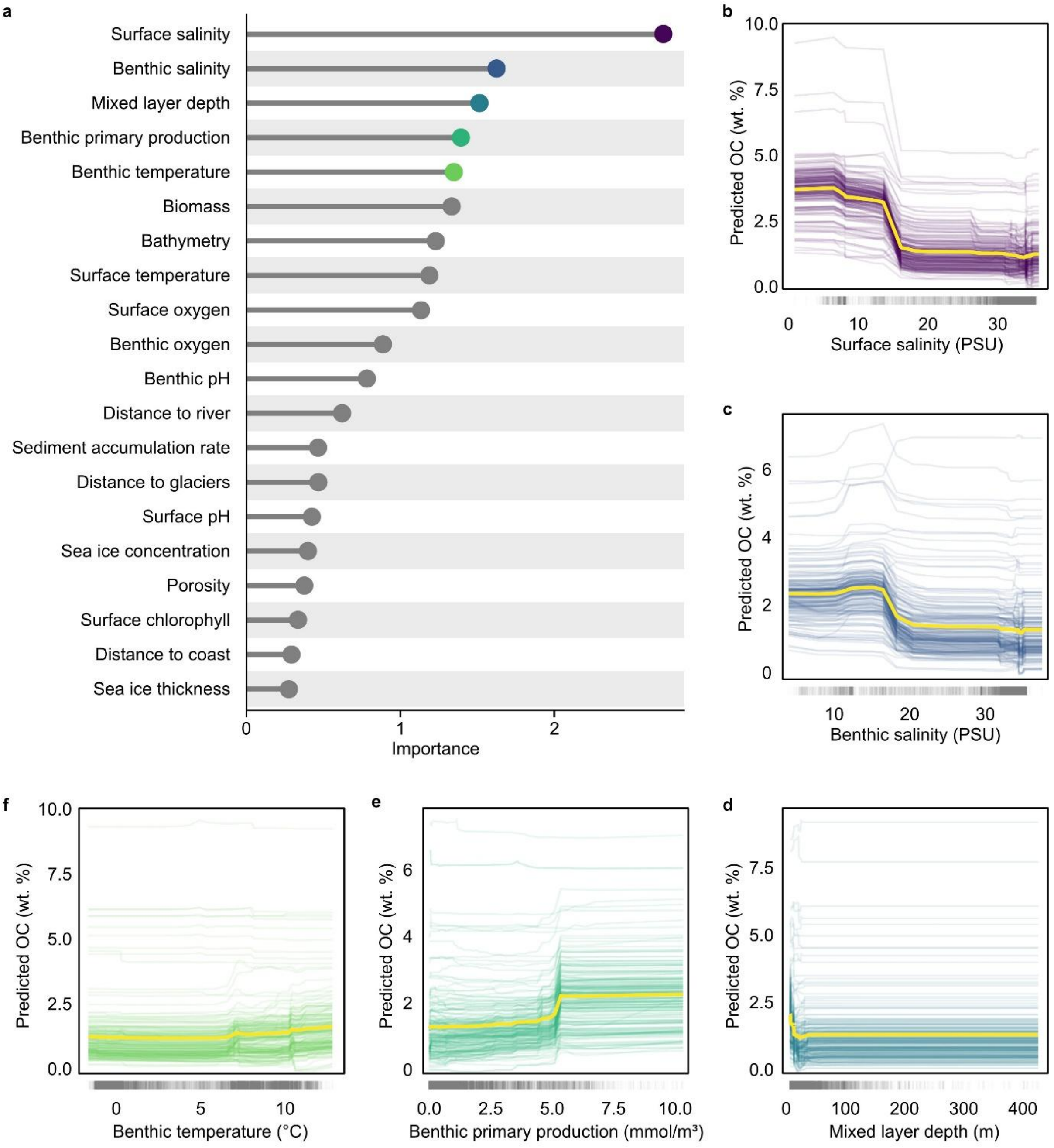

Figure S3: Predictor importance and partial dependence for organic carbon (OC) in surface sediments. a) *20 most important predictor variables based on permutation importance for quantile regression forest modelling of OC in surface sediments.* ***b–f)*** *Partial dependence plots for the five most influential predictors. Thick yellow lines show the partial dependence, thin coloured lines represent individual conditional expectation (ICE) curves and grey rug lines indicate the distribution of observed data.*

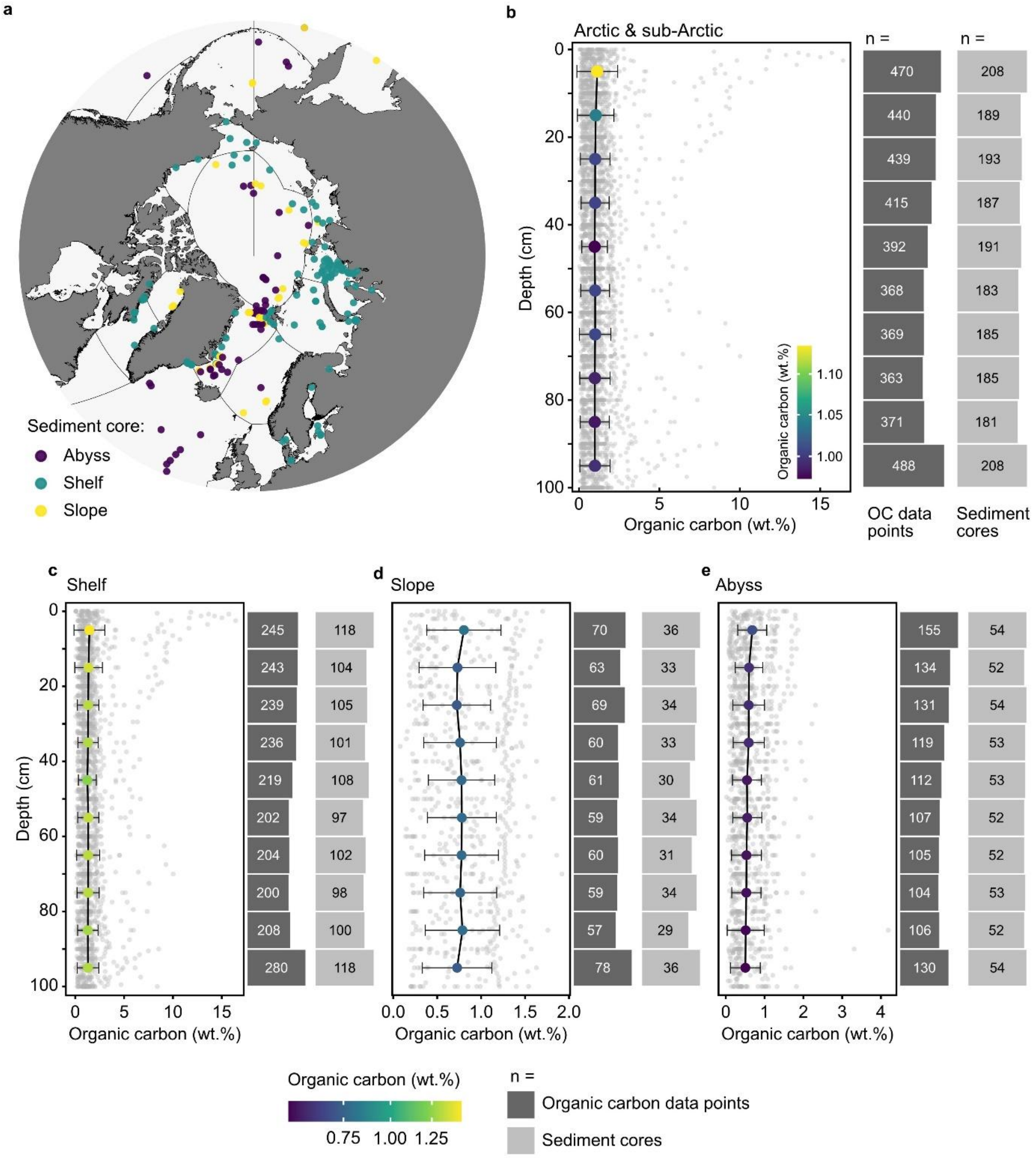

Figure S4: Vertical distribution of organic carbon (OC) in sediment cores across the study region. a) *Map of sediment core locations classified by geomorphic unit.* ***b–e)*** *Mean OC concentrations in the upper 1 m of sediment, derived from sediment core data. Coloured points indicate mean OC concentration (wt.%) for each 10 cm depth interval with horizontal bars indicating ± 1 standard deviation among cores, and grey points represent individual measurements. Histogram plots to the right of each profile show the number of OC measurements (dark grey) and cores (light grey) within each depth interval. Panels show cores from* ***b)*** *shelf,* ***c)*** *slope and* ***d)*** *abyssal regions.*

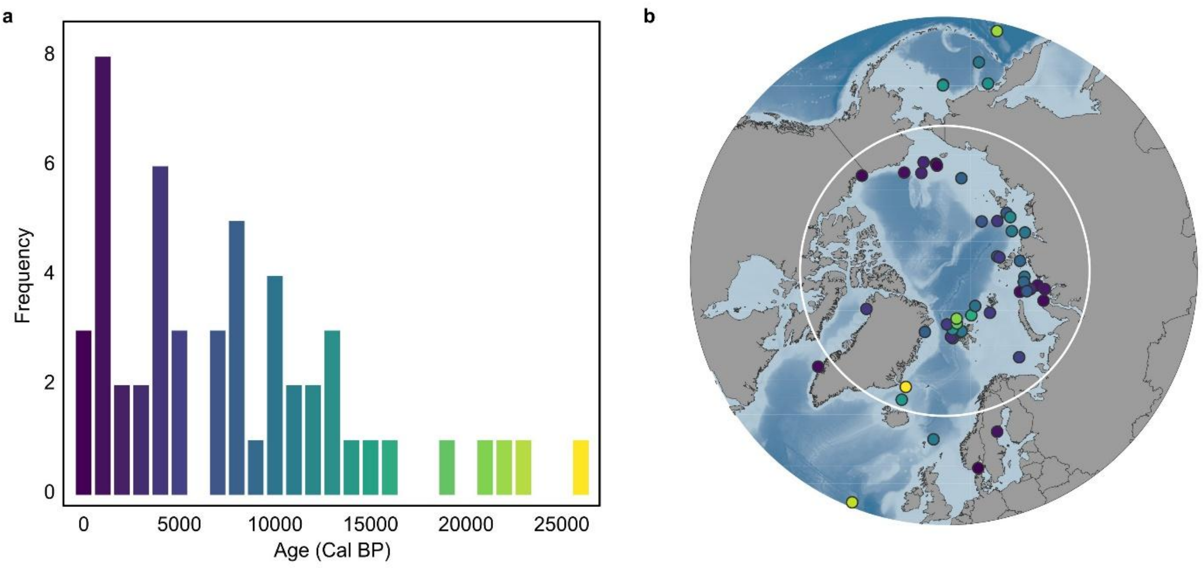

***Figure S5: Age of sediment at 1 m depth. a)** Histogram of age (calibrated years Before Present–cal BP^{1950}) of sediment cores at 1 m depth for cores where radiocarbon dates were available (n = 52). **b)** Distribution of dated cores colour-coded by age in (a), overlaid on a bathymetry chart (International Bathymetry Chart of the Arctic Ocean (IBCAO)[1]). Arctic circle (66°N) delineated by white circle.*

***Table S1: Organic carbon (OC) concentration, accumulation rate (OCAR), depth-integrated OC density and total OC stock in the upper 10 cm of Arctic and sub-Arctic (50–90°N) by geomorphic units.** Mean values represent the average across all raster grid cells within each unit at the median prediction, with values in parentheses indicating the 5th and 95th conditional quantiles.*

	Area (x10^5 km^2)	OC (mean wt.%)	OCAR (mean g m^{-2} yr^{-1})	OC density (mean g cm^{-2})	Total OC stock (Pg)
Shelf	126	0.96 (0.27–2.39)	1.85 (0.44–5.16)	0.10 (0.03–0.24)	12.07 (3.46–29.89)
Slope	31.9	0.70 (0.23–1.65)	0.28 (0.08–0.68)	0.06 (0.02–0.14)	1.94 (0.63–4.60)
Hadal	1.49	0.62 (0.64–1.57)	0.16 (0.04–0.37)	0.04 (0.01–0.09)	0.06 (0.02–0.14)
Abyss	129	0.61 (0.24–1.21)	0.07 (0.03–0.13)	0.04 (0.02–0.09)	5.59 (2.25–11.14)
				**Total:**	19.65 (6.35–45.77)

Supplementary Methods:

S1: Sediment core data collection

Literature search:

A systematic review of literature in Scopus was conducted in October 2023 by constructing a Boolean search string of a combination of keywords from 4 topics (variable, setting, environment, and location) that searched the title, abstract and keywords. This search returned 7786 results. Titles and abstracts were screened to identify publications of interest using ASReview[2]. Briefly, ASReview is an open-source machine learning aided pipeline that applies active learning to improve the efficiency of reviewing literature by reducing the number of records that require manual screening. Prior knowledge is selected by the user which is used to train the first model. In this case, 50 relevant records were identified and labelled, and 50 labelled as irrelevant. An active learning cycle then begins whereby a new record is screened and labelled by the user. The user's label is subsequently used to train a new model, and a new record is presented to the user. The cycle continues until a user-defined stopping criterion has been reached. In this study, 3000 papers were screened at which point the prior 168 records had been user labelled as irrelevant. Titles and abstracts of selected relevant publications were then screened which identified 293 relevant articles.

Dataset search:

A search of open-source data publishers and online cruise reports was conducted. In June 2023, a search was performed in PANGAEA using the search terms 'marine sediment core organic carbon TOC' and refined to the geographical region of 50–90°N. This returned 1,082 individual datasets which were examined for OC data. This was later (March 2024) completed with cores from the Baltic region. Cruise reports from research expeditions of the Ocean Drilling Program (1985–2003), Integrated Ocean Drilling Program (2003–2013), GEOMAR and JAMSTEC were also consulted for additional cores.

S2: Sediment core database construction

OC content (wt.%) was extracted from publication tables or supplementary information and datasets were downloaded from data repositories. When raw OC% data were not provided in publications or as supplementary material, corresponding authors were contacted. OC data was reported alongside the corresponding core depth. Where data was reported as a depth interval (e.g. 0–1cm), the mid-point was recorded. Total nitrogen (TN) (wt.%), OC/TN ratios (mass or atomic ratios depending on source), $\delta^{13}C$ (‰) and $\delta^{15}N$ (‰) are also reported where

available. Analysis methods of OC and TN and isotopic data have been reported alongside. Storage conditions prior to analysis are also included.

Metadata for all cores was also extracted and recorded (Table S2). Sediment core name, typically in the format “cruise name_core number” is used to ensure a unique identifier for individual cores. In cases where a name was not provided, cores have been assigned an identifier based on the same format.

Spatial information for individual cores is provided in the database. Coordinates of core location are reported in decimal degrees and converted where necessary. In some cases, coordinates were not reported in the literature, and core location was shown on a figure/map. For such instances, coordinates have been estimated and these cores are identified in the ‘Notes’ section. The ocean or sea for which the core was extracted is reported using boundaries defined by the International Maritime Organisation (IMO)[3]. The geomorphic unit (i.e., continental shelf, continental slope, abyss or hadal)[4] of each core is also identified. The database contains a column for water depth as reported by the data source. Where water depth was not provided in the literature or data repository, depth was estimated using the International Bathymetry Chart of the Arctic Ocean (IBCAO; v.4)[1].

Additional metadata includes the coring technology (e.g. gravity corer, drilling), core length alongside the campaign name or number, research vessel from which the core was collected and the year of collection.

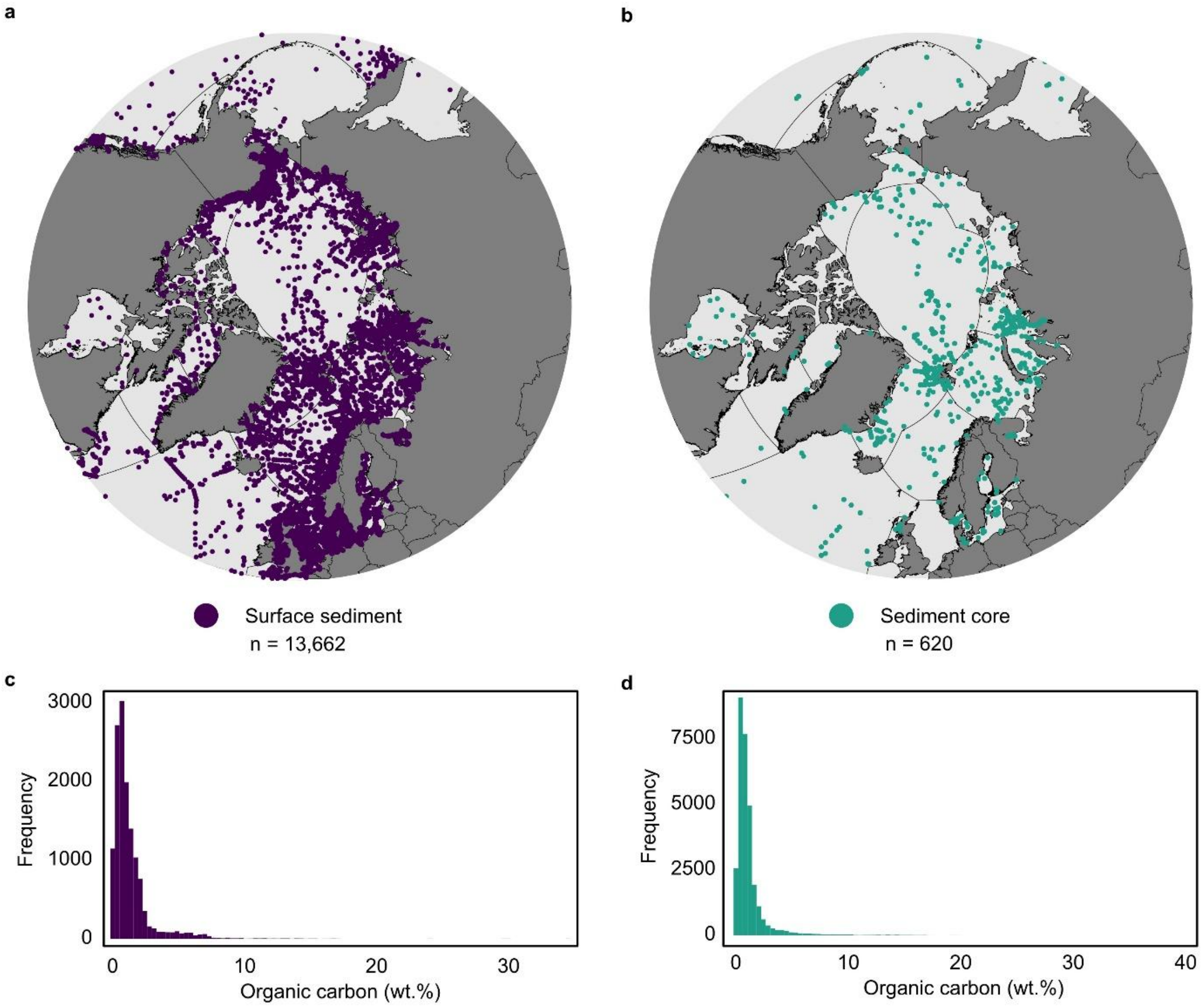

Figure S6: Distribution of organic carbon (OC) data across the Arctic and sub-Arctic. a) Surface-sediment OC measurements used for predicting OC% in surface sediments. **b)** Sediment cores used to characterise downcore OC% variability. c) Histogram of OC% for surface sediment samples. d) Histogram of OC% for sediment cores across all depths.

Table S2: *Description of variables in the database.*

	Variable Name	Units	Description
Metadata	CORE ID		Unique identifier of sediment core
	LAT	Decimal degrees	Latitude
	LON	Decimal degrees	Longitude
	REGION		Sea/ocean of core location as defined by IMO
	GEOMORPHIC		Geomorphic unit of core location as defined by Harris et al., 2014
	WATERDEPTH	Meters below sea level (mbsl)	Water depth of sediment core
	WATERDEPTH IBCAO	Meters below sea level (mbsl)	Water depth of sediment core according to IBCAO
	CORING TECHNOLOGY		Coring technology used to extract core
	CORE LENGTH	Meters (m)	Length of core retrieved
	CAMPAIGN		Research campaign name
	RESEARCH VESSEL		Research vessel name
	YEAR		Year of sediment core extraction
	DEPTH	Centimetres (cm)	Mean depth of sample
Geochemistry	OC (%)	%	Total organic carbon concentration of the bulk sediment
	TN (%)	%	Total nitrogen concentration of the bulk sediment
	OC:TN		Total organic carbon to total nitrogen ratio (reported as mass or atomic ratios depending on source)
	δ^{13}C (‰)	‰	^{13}C content
	δ^{15}N (‰)	‰	^{15}N content
Geochemistry methods	STORAGE		Storage method before organic carbon analysis; 0: unknown, 1: frozen, 2: refrigerated, 3: room temperature
	OC/TN METHOD		Method of organic carbon and total nitrogen analysis
	δ^{13}C/δ^{15}N METHOD		Method of isotopic analysis
Chronologies	LAB CODE		Laboratory code for radiocarbon age
	MATERIAL		Material used for dating
	14C AGE (yr BP)		Uncalibrated radiocarbon age
	ERROR (yr)		Uncertainty of uncalibrated radiocarbon date
	RESEVOIR EFFECT		Reservoir effect applied for age calibration
	RESEVOIR EFFECT ERROR		Reservoir effect error applied for age calibration
	CAL AGE μ (yr BP)		Mean calibrated radiocarbon age
	CAL AGE σ (yr BP)		Uncertainty of calibrated radiocarbon age
	CAL AGE MEDIAN (yr BP)		Median calibrated radiocarbon date
Citati ons	OC/TN CITATION		Citation for organic carbon and total nitrogen analysis
	δ^{13}C/δ^{15}N CITATION		Citation for isotopic analysis

	CHRONOLOGY CITATION		Citation for chronology information
	ADDITIONAL CITATION		Any additional associated citations
	NOTES		Notes

Table S3: Description of predictor variables used in the quantile regression forest model. *Astrix (*) designates those that were not used in the final model due to high correlation. GEBCO: General Bathymetric Chart of the Oceans; GLIMS: Global Land Ice Measurements from Space. ESA: European Space Agency.*

Category	Variable	Native spatial resolution	Temporal resolution	Processing	Source
Sediment & seafloor properties	Sediment accumulation rate	2 arc-min	-	Reprojection, resample & spatial crop	[5]
	Porosity	5 arc-min	-	Reprojection, resample & spatial crop	[6]
	Biomass	5 arc-min	-	Reprojection, resample & spatial crop	[7]
	Bathymetry	15 arc-second	-	Reprojection, resample, spatial crop & mask positive (land) values	GEBCO[8]
Water column parameters	Surface and benthic temperature	5 arc-min	Mean 2000-2019	Reprojection, resample & spatial crop	Bio-ORACLE[9,10]
	Surface and benthic salinity	5 arc-min	Mean 2000-2019	Reprojection, resample & spatial crop	Bio-ORACLE[9,10]
	Surface and benthic dissolved oxygen	5 arc-min	Mean 2000-2018	Reprojection, resample & spatial crop	Bio-ORACLE[9,10]
	Surface and benthic pH	5 arc-min	Mean 2000-2018	Reprojection, resample & spatial crop	Bio-ORACLE[9,10]
	Surface chlorophyll	5 arc-min	Mean 2000-2018	Reprojection, resample & spatial crop	Bio-ORACLE[9,10]

	Surface* and benthic primary production	5 arc-min	Mean 2000-2020	Reprojection, resample & spatial crop	Bio-ORACLE[9,10]
	Mixed layer depth	5 arc-min	Mean 2000-2019	Reprojection, resample & spatial crop	Bio-ORACLE[9,10]
	Surface and benthic current velocity	5 arc-min	Mean 2000-2019	Reprojection, resample & spatial crop	Bio-ORACLE[9,10]
Sea ice	Sea ice concentration	5 arc-min	Mean 2000-2020	Reprojection, resample & spatial crop	Bio-ORACLE[9,10]
	Sea ice thickness	5 arc-min	Mean 2000-2020	Reprojection, resample & spatial crop	Bio-ORACLE[9,10]
Spatial	Distance from coast	1 km	-	Euclidean distance calculated & resample	World Countries Generalised[11]
	Distance from rivers (discharge >100 $m^3 s^{-1}$)	1 km	-	Euclidean distance calculated & resample	HydroRIVERS[12]
	Distance from glaciers	1 km	-	Euclidean distance calculated & resample	GLIMS[13]
	Distance from permafrost*	1 km	-	Euclidean distance calculated & resample	ESA Permafrost Climate Change Initiative[14]

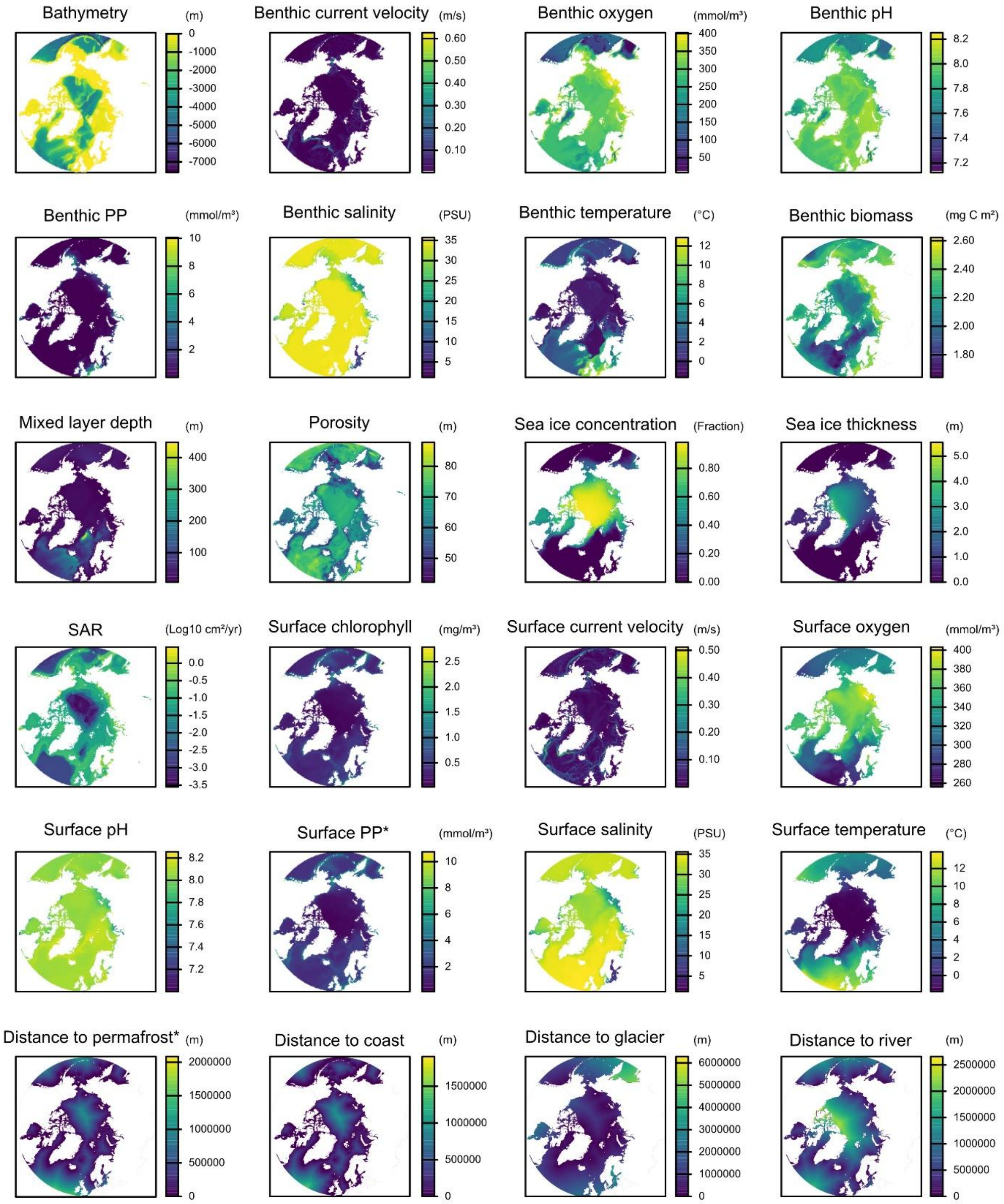

Figure S7: Predictor variables used in the random forest model. *Variables marked with an asterisk (*) were excluded from the final model due to high collinearity identified during variable selection. PP, primary production; SAR, sediment accumulation rate.*

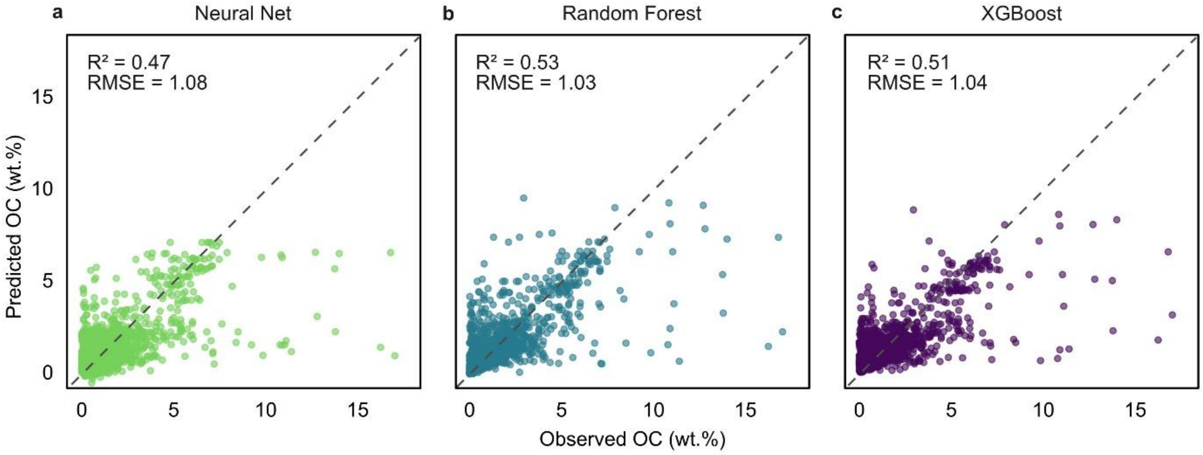

***Figure S8: Performance of the machine learning algorithms tested to predict organic carbon (OC) in surface sediments**. Observed and predicted OC from **a)** neural net, **b)** random forest and **c)** XGboost modelling. Points represent data withheld from the model and dashed grey line represents the 1:1 ratio where observed value is equal to predicted value. R^2 and RMSE for each model is shown as a measure of performance.*

Supplementary Material Reference List

1. Jakobsson, M. *et al.* The International Bathymetric Chart of the Arctic Ocean Version 4.0. *Sci. Data* 7, 1–14 (2020).

2. van de Schoot, R. *et al.* An open source machine learning framework for efficient and transparent systematic reviews. *Nat. Mach. Intell.* 3, 125–133 (2021).

3. Flanders Marine Institute. IHO Sea Areas, version 3. https://www.marineregions.org/ (2018) doi:https://doi.org/10.14284/323.

4. Harris, P. T., Macmillan-Lawler, M., Rupp, J. & Baker, E. K. Geomorphology of the oceans. *Mar. Geol.* 352, 4–24 (2014).

5. Restreppo, G. A., Wood, W. T., Graw, J. H. & Phrampus, B. J. A machine-learning derived model of seafloor sediment accumulation. *Mar. Geol.* 440, 106577 (2021).

6. Martin, K. M., Wood, W. T. & Becker, J. J. A global prediction of seafloor sediment porosity using machine learning. *Geophys. Res. Lett.* 42, 10640–10646 (2015).

7. Lee, T. R., Wood, W. T. & Phrampus, B. J. A Machine Learning (kNN) Approach to Predicting Global Seafloor Total Organic Carbon. *Global Biogeochem. Cycles* 33, 37–46 (2019).

8. GEBCO Compilation Group. GEBCO 2023 Grid. https://www.gebco.net/data-products/gridded-bathymetry-data (2023) doi:10.5285/37c52e96-24ea-67ce-e063-7086abc05f29.

9. Tyberghein, L. *et al.* Bio-ORACLE: a global environmental dataset for marine species distribution modelling. *Global Ecology and Biogeography* 21, 272–281 (2012).

10. Assis, J. *et al.* Bio-ORACLE v2.0: Extending marine data layers for bioclimatic modelling. *Global Ecology and Biogeography* 27, 277–284 (2018).

11. ESRI, Garmin International, Inc., The U.S. Central Intelligence Agency & National Geographic Society. World Countries Generalized. Preprint at https://services.arcgis.com/P3ePLMYs2RVChkJx/arcgis/rest/services/World_Countries_(Generalized)/FeatureServer (2022).

12. Lehner, B. & Grill, G. Global river hydrography and network routing: Baseline data and new approaches to study the world’s large river systems. *Hydrol. Process.* 27, 2171–2186 (2013).

13. GLIMS & NSIDC. Global Land Ice Measurements from Space glacier database. Compiled and made available by the international GLIMS community and the National Snow and Ice Data Center, Boulder CO, U.S.A. https://doi.org/10.7265/N5V98602 (2005) doi:10.7265/N5V98602.

14. Westermann, S. *et al.* ESA Permafrost Climate Change Initiative (Permafrost_cci): Permafrost version 4 data products. *NERC EDS Centre for Environmental Data Analysis* https://catalogue.ceda.ac.uk/uuid/c116848868054e18a9f5a51d68c3ad21. (2025).
