# Supplementary material for: Hotspots of Arctic and sub-Arctic marine sediment organic carbon are dominated by the Baltic, Barents and Chukchi Seas
Source: Commun Earth Environ. 2026 Jun 19;7(1):529. doi: 10.1038/s43247-026-03720-8 (PMC13278947; doi:10.1038/s43247-026-03720-8)
Supplement: Supplementary file 1 — Transparent Peer Review file [file 43247_2026_3720_MOESM1_ESM.pdf]

nature portfolio

Peer Review File

Hotspots of Arctic and sub-Arctic marine sediment organic carbon are dominated by the Baltic, Barents and Chukchi Seas

Corresponding Author: Professor Nicholas Kamenos

This file contains all editorial decision letters in order by version, followed by all author rebuttals in order by version.

Version 0:

Decision Letter:

Dear Professor Kamenos,

Thank you for submitting your manuscript titled "Arctic and sub-Arctic sediments host disproportionate portion of global marine organic carbon stock". Regretfully, we cannot publish it in Communications Earth & Environment.

It is our policy to decline a substantial proportion of manuscripts without sending them to referees, so that they may be sent elsewhere without further delay. Such decisions are made by the editorial staff when papers do not meet the criteria for publication in Communications Earth & Environment.

At this stage we consider, for example, whether a manuscript is likely to be of considerable interest to those working in the same area of the Earth, planetary and environmental sciences and to stimulate new thinking in the field. In this case, we are not persuaded that your findings represent a sufficiently striking advance to justify publication in Communications Earth & Environment. Please note, however, that we do not question the validity of your work.

As a service to our authors, we have also assessed potential suitability for consideration in Communications Sustainability, a new journal from the Nature Portfolio.

Although we cannot offer to publish your manuscript, I suggest that you consider Scientific Reports as a suitable venue for this work. To transfer your manuscript, please use our manuscript transfer portal. You will not have to re-supply manuscript metadata and files, unless you wish to make modifications. For more information, please see our manuscript transfer FAQ page.

We are sorry that we cannot be more positive on this occasion and thank you for the opportunity to consider your work.

Best regards,

Alice Drinkwater, PhD
Associate Editor
Communications Earth & Environment
Consulting Editor
Communications Sustainability

Version 1:

Decision Letter:

** Please ensure you delete the link to your author home page in this e-mail if you wish to forward it to your coauthors **

Dear Professor Kamenos,

Please accept our apologies for the delay in sending a decision on your manuscript. Your manuscript titled "Hotspots of Arctic and sub-Arctic marine organic carbon burial are dominated by the Baltic, Barents and Chukchi Seas" has now been seen by 3 reviewers, and we include their comments at the end of this message. They find your work of interest, but some important points are raised. We are interested in the possibility of publishing your study in Communications Earth & Environment, but would like to consider your responses to these concerns and assess a revised manuscript before we make a final decision on publication.

We therefore invite you to revise and resubmit your manuscript, along with a point-by-point response that takes into account the points raised. Please highlight all changes in the manuscript text file.

Please submit your point-by-point responses as a separate file, distinct from your cover letter where you can add responses to the Editors' comments that you do not want to be made available to the reviewers. Word files are preferred. We recommend that any figures, tables or graphs that are included in the response to reviewers are also included in the main article or Supplementary Information.

We are committed to providing a fair and constructive peer-review process. Please don't hesitate to contact us if you wish to discuss the revision in more detail.

Please use the following link to submit your revised manuscript, point-by-point response to the referees' comments (which should be in a separate document to any cover letter), a tracked-changes version of the manuscript (as a PDF file) and the completed checklist:
Link Redacted
** This url links to your confidential home page and associated information about manuscripts you may have submitted or be reviewing for us. If you wish to forward this email to co-authors, please delete the link to your homepage first **

We hope to receive your revised paper within six weeks; please let us know if you aren't able to submit it within this time so that we can discuss how best to proceed. If we don't hear from you, and the revision process takes significantly longer, we may close your file. In this event, we will still be happy to reconsider your paper at a later date, as long as nothing similar has been accepted for publication at Communications Earth & Environment or published elsewhere in the meantime.

Please do not hesitate to contact us if you have any questions or would like to discuss these revisions further. We look forward to seeing the revised manuscript and thank you for the opportunity to review your work.

Best regards,

Alice Drinkwater, PhD
Associate Editor
Communications Earth & Environment
Consulting Editor
Communications Sustainability

EDITORIAL POLICIES AND FORMATTING

We ask that you ensure your manuscript complies with our editorial policies. Please ensure that the following formatting requirements are met, and any checklist relevant to your research is completed and uploaded as a Related Manuscript file type with the revised article.

For Manuscripts that fall into the following fields:
• Behavioural and social science
• Ecological, evolutionary & environmental sciences
• Life sciences
An updated and completed version of our Reporting Summary must be uploaded with the revised manuscript
You can download the form here:
https://www.nature.com/documents/nr-reporting-summary.pdf

Furthermore, please align your manuscript with our format requirements, which are summarized on the following checklist:
Communications Earth & Environment formatting checklist

and also in our style and formatting guide Communications Earth & Environment formatting guide .

*** DATA: Communications Earth & Environment endorses the principles of the Enabling FAIR data project (http://www.copdess.org/enabling-fair-data-project/). We ask authors to make the data that support their conclusions available in permanent, publically accessible data repositories. (Please contact the editor if you are unable to make your data available).

All Communications Earth & Environment manuscripts must include a section titled "Data Availability" at the end of the

Methods section or main text (if no Methods). More information on this policy, is available at http://www.nature.com/authors/policies/data/data-availability-statements-data-citations.pdf.

In particular, the Data availability statement should include:
- Unique identifiers (such as DOIs and hyperlinks for datasets in public repositories)
- Accession codes where appropriate
- If applicable, a statement regarding data available with restrictions
- If a dataset has a Digital Object Identifier (DOI) as its unique identifier, we strongly encourage including this in the Reference list and citing the dataset in the Data Availability Statement.

DATA SOURCES: All new data associated with the paper should be placed in a persistent repository where they can be freely and enduringly accessed. We recommend submitting the data to discipline-specific, community-recognized repositories, where possible and a list of recommended repositories is provided at http://www.nature.com/sdata/policies/repositories.

If a community resource is unavailable, data can be submitted to generalist repositories such as figshare or Dryad Digital Repository. Please provide a unique identifier for the data (for example a DOI or a permanent URL) in the data availability statement, if possible. If the repository does not provide identifiers, we encourage authors to supply the search terms that will return the data. For data that have been obtained from publically available sources, please provide a URL and the specific data product name in the data availability statement. Data with a DOI should be further cited in the methods reference section.

Please refer to our data policies at http://www.nature.com/authors/policies/availability.html.

REVIEWER COMMENTS:

Reviewer #1 (Remarks to the Author):

The study of Langley et al. presents sedimentary OC% and OCAR in the Arctic and sub-Arctic based on an impressive dataset, which is spatially upscaled using ‘Quantile Regression Forest (QRF)’ modelling. The distributions reveal spatial heterogeneities across the study region, highlighting OC% and OCAR hot- and coldspots, and the authors discuss different potential driving factors for their observations. Furthermore, the spatial distribution is used to quantify carbon stocks and highlight the study region as an important organic carbon sink in a global context. In that, the study provides important and novel results to environmental and climate scientists, especially since the high latitudes are comparably understudied regions and are associated with relatively high uncertainties in previous global model assessments. The methods for data gathering, spatial upscaling and calculations are state-of-the-art, well validated and provide robust results. Regarding relatively high model uncertainties, it is important to consider that this is typical for spatial upscaling, with QRF modelling generally recognised as one of the best performing methods. Figures and tables are well-designed and informative. Nevertheless, I think the manuscript can benefit from major improvements before publication. My main critiques are (1) confusing/wrong terminologies and units, (2) reproducibility of the methods, and (3) interpretation of predictors as key drivers. I address these and additional remarks below.

Considering the robust methodology, relevance to the field, and the areas identified for improvement, I would recommend a Major Revision.

Line 33: I suggest mentioning QRF as the applied method to spatially upscale the ~14000 datapoints in the abstract.

Line 52: Please double-check the bibliography of the first six citations, as they seem to be wrongly attributed in the manuscript.

Line 53-57: I suggest rewriting: “The biological carbon pump, riverine inputs, coastal erosion and glacial discharge deliver OC to the ocean floor, a proportion of which is buried in sediments over millennia”. For these broad statements, I suggest citing more general previous work, such as Berner et al. (1980), Early diagenesis: a theoret. approach, and cut the reference to fjords, as they are not a specific focus of this study.

Line 64-66: I am not sure if I understand this statement correctly. If we have an understanding of processes contributing to OC accumulation and burial at high latitudes, I assume these processes also contribute to the creation of hot- or coldspots? Consider rewriting. Furthermore, here the terms “accumulation” and “burial” are introduced and I suggest phrasing clear definitions. OC accumulation is the deposition and storage of OC at certain depths (here defined as up to 10 cm sediment depths). OC burial is the long-term storage (calculated at depth below which OC% stays near constant). OC deposition flux/rain rate is the OC flux that reaches the seafloor. I suggest to critically review the usage of these terms throughout the manuscript and adapting them with respect to the definitions set in the introduction.

Line 82-83: Consider citing Zhang et al. (2024; https://doi.org/10.1038/s41561-024-01581-4), showing that bottom trawling impact relates to OC contents in the North Sea.

Line 93-95: Consider citing Diesing et al. (2024) https://doi.org/10.1038/s43247-024-01502-8.

Line 108: What general lengths do the sediment cores have? E.g. "sediment cores (> 50 cm)".

Line 116: spatially

Line 172: "This likely reflects a combination of remineralisation with OC age in deeper sediments". I would assume that deposited OC in the Barents and Chukchi Seas at greater water depths will be more degraded compared to the shallow Baltic Sea. Consequently, I would expect lower degradation rates of the already refractory OC in sediments of the Barents and Chukchi Seas. The argument of lower starting or "initial" OC concentration makes sense and could be the sole argument for explaining why OC concentrations fall below the 1.43 wt% hotspot threshold.

Line 185: Please mention how this finding relates to global surface OC storage (i.e. it is approximately 13% (4-29%) of the global 156 Pg number).

Line 188-191: For densities, I would expect a unit of g/cm3 instead of g/cm2. Given the magnitude of the numbers, I assume the densities were integrated over the top 10 cm of sediments? If so, please describe that the presented numbers are integrated densities and not volumetric densities.

Line 191: A suggestion for rephrasing: The elevated OC stock on shelves underscores their importance for high-latitude sedimentary carbon storage. Notably, OC cycling on shelves is highly dynamic given the proximity to land, with changes in terrestrial carbon inputs and marine primary production rapidly influencing deposition patterns, as well as frequent and intense human disturbances to the seabed.

Line 205: The Arctic has been previously shown as a relatively high OC region, including early work by Berner (1982; https://doi.org/10.2475/ajs.282.4.451) or Hedges and Emerson (1988; https://doi.org/10.1029/PA003i005p00621). Consider citing these early works.

Line 210: I would avoid the term "hotspot" here, as it is defined as being in the top 5% of OC values in this study. A reader could think that the 0.90 wt% Arctic value is in the top 5% of global OC data. I suggest to write "important region in a global context".

Line 224-226: I find the argumentation about chronology a bit confusing. A temporal analysis (e.g. OC stock generation in the last x years across a region) seems not to be the scope of the sentences in lines 217-224 and of this study in general. I suggest to rephrase the text to "These results suggest that extrapolating surface sediment OC concentrations to deeper sediment layers would introduce large uncertainty in OC stock quantifications.", as this highlights the continuous decrease of OC% with depth below 10 cm at many sites.

Line 229 - 244: I suggest to restructure this paragraph and add an explanation to the predictive capability of salinity: "High OC% occurs close to land, with the most pronounced hotspots in the semi-enclosed, shallow Baltic Sea (including the Gulf of Finland and Gulf of Bothnia). Here, restricted water exchange with the open ocean and freshwater discharge from surrounding landmasses lead to a pronounced vertical stratification of the water column, creating environmental conditions favourable for OC accumulation35. These stratified conditions likely promote bottom-water hypoxia, suppressing benthic remineralisation and bioturbation and enhancing OC preservation36. Consistent with this, surface and benthic salinity ranked as the highest-importance predictors of OC% (Fig. S2a), with non-linear increases in predicted OC% at lower salinities (Fig. S2b, c). However, it is important to note that salinity itself does likely not have a direct effect on OC% or OCAR, but relates to environmental conditions, such as stratification and hypoxia, that drive OC preservation. Mixed layer depth was another important predictor that is associated with water column stratification, with higher OC concentrations at shallower mixed layers (Fig. S2d). In addition to the stratification effect, a shallow mixed layer can concentrate nutrients and phytoplankton biomass within the euphotic zone, enhancing primary production and the efficiency of OC export to depth37. A further reason for particularly high OC% in the Baltic Sea is a long history of human activities, including sustained nutrient loading, elevating primary production and sedimentary OC inputs35, in contrast to higher-latitude Arctic shelves where anthropogenic influence is weaker and OC% is generally lower."
I believe it is important to clarify that there is likely no direct effect of salinity on OC preservation. The salinity gradient (not absolute salinity) results in stratification and induces deep water hypoxia, which in turn drives OC preservation. Since the presented predictor is salinity, not salinity gradient (i.e. difference between surface and bottom waters), it may be confusing to the reader to say "salinity drives OC preservation".

Line 256: The Mackenzie River and the Lena River

Line 259-263: "Consistent with this interpretation of our findings". Consider shortening this to "Consistent with this interpretation". If the authors have data on (long) sediment cores from Arctic coastline areas, it would be interesting to compare the transfer efficiency (Teff) of these areas to the 62% and 77% of the Chukchi and Barents Sea, respectively. If it is old, refractory OC that is deposited along the Arctic coastline hotspots, then it should be reflected as a higher transfer efficiency than the areas with high inputs of fresh marine material, which could strengthen this interpretation.

Line 271: Consider citing Zhang et al. (2024); https://doi.org/10.1038/s41561-024-01581-4.

Line 275-278: This reads a bit confusing and could need a bit more explanation, especially the "different sediment ages at

equivalent depths down core" part in context to OCAR and OC burial being different. OCAR and OC burial can be different from one another, irrespective of age, OC input or sedimentation rates (depending on how they are defined, see line 64). In other words, even if you had a temporal analysis, OCAR and OC burial could still be different.

Line 282: How does the mismatch in OCAR vs OC% in Fig. 3 translate to OCAR being different from OC burial? OCAR is different from OC burial because of continuous OC remineralisation below 10 cm (which is the cut-off point for OCAR calculations in this study). Please explain or rewrite the sentence.

Line 283: Compiled.

Line 293: The unit of sedimentation rates should be in cm yr-1.

Line 304-305: Consider rewriting this sentence to: Hence, OC may reach marine sediments at an already old and refractory state in river-dominated high Arctic areas, which could partly explain the large range of radiocarbon-derived ages we observed at 1 m sediment depth (Fig. S4).

Line 316: This paragraph seems a bit loose/repetitive to text from above and is not well-connected to the general findings of this study. For example, the study does not show that source-to-sink transits are particularly important in the Arctic compared to other regions globally. Conversely, the study highlights other processes to be important in controlling the distribution of OC% and OCAR, such as the salinity anomaly in the Baltic Sea (and related environmental conditions). Anthropogenic disturbances seem to be another important factor. While source-to-sink factors, such as primary production and riverine inputs, are also mentioned, I am not sure whether this justifies its own paragraph. Especially since the source-to-sink relation and the importance of the highly dynamic shelf areas for OC% and OCAR have been mentioned in other parts of the manuscript. I suggest to merge text compartments of this paragraph with the section 'Bio-environmental controls on organic carbon hotspot creation'.

Line 335-338: Regarding the freshening trend of Arctic surface waters and the resulting strengthening of water column stratification in the future: I suggest adding here that contemporary Baltic Sea conditions (enhanced salinity gradient, stratification, hypoxia, nutrient load, etc.) could in part reflect how other Arctic ocean regions may evolve in the future. This also nicely follows up on the idea of Reusch et al. (2018), who highlight the Baltic Sea as 'a time machine for the future ocean' (DOI: 10.1126/sciadv.aar8195).

Line 338-354: These text parts read more like a general introduction, as they summarize the effects of warming on the Arctic in general, but they do not directly connect to the main findings of this study. I suggest moving a condensed version of it to the introduction after line 63.

Line 370-383: "Even though the impact of physical disturbance on sediment OC stocks remains debated (e.g.21–24), using the precautionary principle there is a need to consider whether protection now of Arctic and sub-Arctic carbon stocks or post-damage restoration (if later deemed necessary) is a better option, given other socio-ecological factors.". I think this text part reads a bit loose and I suggest to merge it with the line above to: "However, such disturbances are projected to increase as a result of diminishing sea ice and a warmer Arctic Ocean25. Given that coastal management at lower latitudes has proven effective in protecting carbon stocks70, there is a need to consider similar strategies for Arctic and sub-Arctic carbon stocks. Based on our assessment of OC% and OCAR hotspots (Fig. 3), we have identified two areas for primary consideration in this context:

1. The high Arctic continental shelf, storing approximately xxx PG of old and young OC that may be vulnerable to future anthropogenic disturbances.
2. Sub-Arctic hotspots for OC% and OCAR, storing approximately xxx Pg of predominantly young OC, yet ongoing and frequent human disturbances diminish their potential to contribute to atmospheric CO2 removal."

Line 385: For the conclusion, I suggest mentioning the total organic carbon stock estimate for the Arctic + sub-Arctic region (19.65 Pg) and how this number relates to global estimates of 156 Pg (i.e. it is an important region because it stores ~ 13% of global surface sedimentary OC). This should also be included in the organic stock section (Line 184) so that it is not mentioned for the first time in the conclusion.

Line 390: "Key controls on this process are salinity, mixed layer depth and benthic primary production explain the variability in OC% across the region, highlighting the complexity of OC burial dynamics." A word is missing in this sentence (a "which" before "explain"?). Furthermore, as written above, I suggest to formulate interpretations of relationships between predictors and OC contents based on the predictive performance a bit more carefully. While a high predictive performance means that the predictor explains the variability of OC contents well, it does not necessarily establish a causal relationship between predictor and response variable. For instance, salinity is the predictor with the highest predictive performance of the final QRF model results. However, there is (likely) no direct control of salinity on OC accumulation, but rather that salinity gradients indicate the presence of environmental conditions that could be favourable for OC accumulation, which in this study mostly refers to the special conditions in the Baltic Sea (which is a salinity anomaly in the study region) or mass accumulation rates at river mouths (which is not discussed in context to salinity in this study though). This would also better fit with the wider literature, showing correlations and (partly) direct influence between environmental factors like oxygen, sedimentation rate or grain size with OC% across different regions on the smaller and larger scale. Hence, I suggest to formulate it more generally. For example: "Localised environmental conditions, such as pronounced salinity gradients in the Baltic Sea and elevated particle inputs at river mouths, strongly influence OC% and OCAR and drive the substantial spatial variability across the study region, highlighting the complexity of benthic OC cycling.

Line 393: For sub-Arctic regions, the discussion has been started already (as cited publications show). Please rephrase this sentence accordingly.

Line 394: Delete the word “include”?

Line 436: I would suggest renaming the paragraph header to “Predictor variables” or “Predictor variables for spatial OC upscaling” since the chosen predictors do not necessarily control OC% or OCAR. They are self-selected (likely limited by what is available in the literature) and have a certain spatial resolution, here 10km. Meaning that finer-scaled features/processes, such as bathymetry (e.g. smaller seafloor depressions), may not be well reflected by the predictor resolution. Further, as written above, a high predictive performance does not always translate to a “control” of the predictor on the response variable.

Line 450: Is the QRF model based on a previously published R-script? If so, please cite it. If not, I would suggest including the model script for better reproducibility of the results.

Line 451: The more widely used term for this model is “Quantile regression model”. I would suggest using this term throughout the text.

Line 461-463: Please describe the other applied methods in more detail and how they have been implemented (i.e. several neural network and gradient boosting algorithms exist). Alternatively, cite previous applications of QRF models (Diesing et al. (2024), https://doi.org/10.1038/s43247-024-01502-8; Spiegel et al. (2024), 10.3389/fmars.2023.1141448; Friedland et al. (2023), https://doi.org/10.3390/w15183235) to show that QRF is a generally successful method. Friedland et al. (2023) included a comparison between different upscaling techniques in the Baltic Sea, with QRF models showing the best performances.

Line 463: I suggest deleting “out-of-the-box”.

Line 464: I assume it should say high R2 and low RMSE? This would also be consistent with Fig. S7. Otherwise, please revisit the calculations.

Line 496: I suggest referring to a publication introducing the calculation of permutation-based variable importance.

Line 506-507: MAR and OCAR units should be g cm-2 yr-1, i.e. grams per square cm per year.

Line 529: OC stock unit should be g cm-2.

Line 542: A pixel or grid cell is 10 km x 10 km or 10 km2.

Line 592: I suggest using “mass accumulation rates” here rather than “sediment accumulation rates” (which could be mistaken with sedimentation rates), as this follows the calculation in Eq. 1 and the cited literature.

Line 894: Only the top five (not six) predictors are depicted.

Line 898: Are the findings of Figure S3 b and c not very similar to the results from the transfer efficiency calculations? If so, one could show the transfer efficiency here rather than introducing a second way of calculating the difference between surface and deep sediment OC%.

Line 942: Please explain how a percentage over 100% can be interpreted in the figure description, e.g. OC deposition may have been higher, or OC composition has been different in the past compared to more recently deposited sediments, post-depositional alterations, etc.

Reviewer #2 (Remarks to the Author):

Langlet et “Hotspots of Arctic and sub-Arctic marine organic carbon burial are dominated by the Baltic, Barents and Chukchi Seas” reports on carbon stocks and accumulation rates across the Arctic and sub-arctic seas. I have previously reviews this manuscript and the current form is much improved and an important contribution to ongoing effort to map carbon across our seabed.
The surface carbon stock estimates follow a tried and tested approach and produce solid estimates. The interesting portion of the paper is determining the stock to depth (1m) and the accumulation rates.

My only query for the authors is focused on the organic carbon accumulation results. It is unclear how the authors have dealt with non-accumulating areas of the seabed. There are multiple areas in arctic and sub-arctic that do not accumulate or are that are actively being eroded. In other research in the North Atlantic/Arctic an area of applicability approach has been taken to stop the model trying to predict values in non-depositional areas.

https://bg.copernicus.org/articles/22/5975/2025/
https://iopscience.iop.org/article/10.1088/2515-7620/ae14bc

https://www.nature.com/articles/s43247-024-01502-8

Additionally, there is some discussion of human disturbance, but this does not really come to anything. I would remove this and focus on the mapping elements of the paper.

For the hotspot analysis it would be good to reference https://iopscience.iop.org/article/10.1088/2515-7620/ae14bc

Reviewer #3 (Remarks to the Author):

Review of Communications Earth & Environment manuscript entitled "Hotspots of Arctic and sub-Arctic marine organic carbon burial are dominated by the Baltic, Barents and Chukchi Seas" by Langley, B., Burdett, H.L., Cameron, K., Juul-Pedersen, T., Rouillard, A., Slaymark, C., Kamenos, N.A.

Synopsis

In this paper, the authors report a study on the identification of organic carbon (OC) burial hotspots and coldspots in the (sub-)Arctic Ocean based on the analysis of more than 14,000 sediment samples. Using machine learning, they identify the Baltic, Barents and Chukchi Seas as the dominant high latitude marine OC hotspots, and list the factors that control OC burial in these hotspots vs. coldspots. Finally, they also evaluate the sensitivity of these hotspots and OC transit pathways to anthropogenic disturbances and identify priority regions for future carbon management strategies. The found that a high quantity of OC (>17 Pg) is at risk now and in the future owing to these disturbances.

General comments

This an interesting and timely study designed to provide background information on the importance and sensitivity of OC burial hotspots to climate change and anthropogenic activities. It makes use of data available in the literature to try extracting a general picture of OC burial in these critical areas. The study is well designed, and it is presented clearly and efficiently (although with a lot of small mistakes listed in the specific comments, which suggest that the final version of the paper was prepared with haste). It is obvious that the manuscript is the result of an immense amount of work. The writing and visuals are of excellent quality. While this type of study is much needed in these times of climate change and anthropogenic forcing on the organic carbon cycle in marine sediments, I have several concerns with this version of the manuscript as listed in the general and specific comments below.

As a first and foremost, the authors should have mentioned in the title and/or the abstract (plus in the list of keywords) that they used machine learning to identify the hotspots and highlight the relative contribution of predictor variables to OC burial. Machine learning is central to this paper and is thus an element that should be rigorously evaluated by someone with the right expertise. I am not an expert in machine learning and therefore I would not have accepted to review this manuscript if I had known that the authors' conclusions were based almost exclusively on machine learning. When contacted by associate editors, potential reviewer have to decide whether they accept to review a manuscript based on the title and abstract only – it is thus essential that the abstract reflects the content of the paper, including the methodological approach.

An important concern with modelling studies is the quality of the input data. Here, the authors have no control on data quality since it was acquired by many different researchers/technicians in lab from all over the world – were all the data included without discrimination? Was an effort made to weed out the apparent bad data? Identifying the obvious outliers (and describing the criteria used to identify them) would allow not only to eliminate the badly measured OC percentages, but also the samples collected in a deposition environment widely different from the majority of the sampling locations in a given area (see below for more on this point).

Another concern is the use of the word uncertainty to represent the range of possible wt % OC and OCAR values between the 5th and the 95th quantiles. A different qualifier for this range should be used in place of uncertainty since the latter means something very different in statistics. In the Supplementary Material section, the authors use "confidence intervals" for the 5th and 95th limits (not the difference between the two), which is much more accurate than using the word uncertainty as done in this work.

Also, there is no comment/discussion on the precision of their model output aside from providing the R2 and RMSE values in the supplementary material. Yet, non-specialists probably do not know whether the values obtained for the Random Forest model represent a good fit of the modeled values to the measured ones. A quick look at the deviations from the 1:1 line on Figure S7 suggests that the fit is not terribly good – is it? These points should definitely be discussed in the paper to provide the readers with a reasonable idea of the quality of the model outputs.

Linked to the point above, the authors should absolutely explain why they have not taken into account the uncertainties associated with the sediment accumulation rates (SAR) taken from Restreppo et al. in their model development. This is particularly important since Restreppo et al. specifically mention that their "estimates for the mass of glaciated/ice rafted sediments exported to the oceans are not precise", a statement that should raise a red flag when their modelled data is used in (sub-)Arctic environments.

While the list of predictor variables may look complete to the non-specialist, it is missing a critical control of OC preservation

in marine sediments: the effect of minerals on the wt.% values. One the one hand, mineral matter dilutes to varying extents the organic content in a sample, which influences the resulting wt.% OC values. Because mineral dilution is somewhat taken into account through the use of SAR, OCAR is a much more solid proxy for the identification of hot or cold spots compared to wt.% OC. On the other hand, minerals provide physical protection against OC remineralization in the sediment. Iron hydroxides are particularly critical in that respect: through direct interaction with iron hydroxides, between 10-50% of total sedimentary OC (rough numbers) has been shown to be protected from mineralization. It would have been easy to add reactive iron as a predictor variable in the input data since more and more studies report this value. Clays are also providing protection against OC remineralization, although I suspect that clay content is not a variable that is often reported in the literature (not to mention that not all types of clays are equal in this respect).

Because of these concerns (above and below), this paper should be accepted with major modifications only is a machine learning specialist evaluates favorably this contribution.

Specific comments

Line 116: “spatial” should be “spatially”.

Lines 119-120: Please see comment below (comment referring to lines 492-493) regarding the definition for uncertainty used here. What is labelled “uncertainty” in this work is a range of possible values for the samples that are not tagged as coming from a hotspot (>95th quantile) or a coldspot (<5th quantile). I agree with the way this range is presented in the next paragraph (5th-95th conditional quantiles) as this terminology much better represents what these values mean.

Line 125 and elsewhere in the text: The double parentheses are confusing. Rather than inserting the ranges between the medians and the units, I suggest listing them separately since word count is not a critical issue for Comms. Earth & Environ.

Lines 121-141: None of these locations are indicated on the maps. Not every reader is an expert in Geography or Oceanography. Please find a way to indicate them on the maps (or on an additional figure in the Supplementary Material section).

Lines 130-131: Please add the “wt.%” units next to the listed values.

Lines 144-160: The units for OCAR are “g m-2 yr-1”, not “g m2 yr-1”. Please correct everywhere.

Lines 145-146: Remove one of the “and” in the parentheses.

Lines 162-182: None of the calculated efficiencies higher than 100% are discussed here, as they should be. A preservation efficiency higher than 100% either indicate a major change in the OCAR with time (an increase), unreliable data (which brings back the issue of data selection and quality control on the input data used in the model), samples coming from widely varying deposition centres (which is also a problem), or a widely insufficient number of datapoints (which again is a serious problem). These points should definitely be addressed.

Also, lines 162-182: Please see comment on Table S2 (line 942) regarding the uncertainties on transfer efficiencies.

Lines 187-188 and Table S1: The percent contributions should not be listed with two decimal points – this is misleading considering the cumulative uncertainties associated with them.

Lines 190-191 and 216: The units should be “g cm-2”, not “g cm2”.

Line 230: Replace the second “including” by “and”.

Lines 237-238: Not necessarily hypoxia, which is defined with a clear dissolved oxygen concentration threshold. The key factors here are oxygen penetration depth (OPD) and exposure time (OET) in the surface sediment. Simply decreasing dissolved oxygen level concentrations, even when above the defined threshold for hypoxia, has a negative influence on OPD and OET, thus partly reducing the extent of OC remineralization. Please rephrase.

Lines 266-271: This spatial heterogeneity of deposition environments is indeed a major limitation of this work. The sample set used in this work might not reflect the diversity and relative proportion of the different types of deposition environments, which might introduce unquantifiable biases in the model results. While it is impossible to identify and quantify such biases with the available dataset, a warning to this effect should at least be added somewhere within the paper (not in the Supplementary Material). The best place would be just after line 271.

Lines 280-282: This mismatch could also be due to mineral dilution, which have not been taken into account in this work. The most important parameter to identify hotspots is OCSA, not %OC as OCAR is independent of dilution by minerals. This is also why neglecting to consider the uncertainty on the SAR data used in this work (see comment below) is in my opinion a major limitation.

Line 283: “Complied” should be “Compiled”.

Line 293: Why are most of the units wrong?!? Here, “cm2 yr-1” should be “g cm-2 yr-1”. These repeated mistakes cast doubt

on the calculations performed in the model or in the care taken when writing the paper. Also, do you mean 4x10-5 to 10.9x10-5? If yes, then why is there only one sig fig for the lower number of the range and three for the higher one?

Line 295: The separator for the thousands should be a coma, not a space (“26 000” should be “26,000”).

Lines 296-297: Not necessarily. In the Arctic, the reservoir effect (time spent on land before being delivered to and buried in margin sediments) can be very high. This reservoir effect is not accounted for in the way this sentence is written (“buried” should be removed since the age reflects both the reservoir effect and the time the OC was buried in sediments).

Lines 311-312: This sentence is not clear as written. The word “modelled” should be added before “time available” on line 311 (“…affects the modelled time available…”).

Lines 316-325: In fact this change (line 324) is already occurring, which means that the assumption of steady-state SAR and OCSA is shaky at best. These recent changes have most probably affected the model outputs. Given that we have a very good idea of the direction of these changes (as discussed in the following section), the effect of such unaccounted changes on the current model outputs should be mentioned and discussed in this section. The next section only discusses the future potential effects of climate change.

Line 338: The scientific meaning of “Atlantification” should be given for the non-specialist readers.
Lines 358-359: This is a highly debatable conclusion. While it might not help reducing “recent” CO2 emissions, the burial of old permafrost carbon in high Arctic river shelves help reduce current and near future emissions of CO2. Old OC age might not always mean highly refractory OC. Large pools of OC can simply be trapped and shielded from degradation in a frozen compartment, thus aging in situ without its usual concurrent “refractorization”.

Lines 411-435: What was the data quality control framework followed to determine good vs. bad data? Were all of the data included without identifying potential outliers? As you know, the quality of the data available from the literature can be highly variable. Please provide information on this point.

Lines 444-448: Given that the number of datapoints for some of the predictor variables was probably much lower than for other more routinely available, did you evaluate the quality of the 10 km grid reprojections for each predictor? How? Can you provide an assessment of the effect of native data density on the quality of these reprojection, and thus on the modelled influence of these predictor on the identification of OC wt% and OCSA hot/cold spots?

Lines 492-493: While the categorisation of the hotspots (>95th quantile) and coldspots (<5th quantile) makes sense, the definition used in this work for the “uncertainty” as the difference between these two limits does not. What you are defining here is a range of possible values for samples that are neither hot or cold spots. The word uncertainty means something very specific in statistics, and your definition on these two lines does not correspond to the universally accepted meaning of this word. This point raises the issue of uncertainty in the identification of the hotspots and coldspots, which is nowhere to be found in this paper.

Line 506-507 and 511: OCAR units should be “g cm-2 yr-1”, not “g-1 cm2 yr-1”, as they are indicated on Figure 2.

Line 510-511: Why was the uncertainty of the sediment mass accumulation rates of Restreppo et al. not used? Their uncertainties have a significant influence on the quality of the output data from your model given the fact that Restreppo et al. specifically stated the following: “Additionally, the estimates for the mass of glaciated/ice rafted sediments exported to the oceans are not precise”. A reason for not considering this additional source of uncertainty should be provided in the text, and the uncertainties in sediment MAR as calculated in Restreppo et al. should be discussed. It is important to know whether the hotspots calculated here, without taking into account the uncertainties in MAR, correspond to areas of high (or low) MAR uncertainties. These details are important given the approach used to define the hotspots and coldspots in the following paragraph.

Line 529: The units for OC stock should be “g cm-2” rather than “g-1 cm2”.

Lines 533-534: Again, this is not an “uncertainty” as understood by most.

Line 552: Is this a safe assumption (constant sedimentation rate)? Given that the Arctic is the area most affected by global warming, one would expect significant changes in the recent sedimentation rates. Please comment. The assumption of constant bulk density is safer but given the magnitude of the changes affecting the (sub-)Arctic environment, assuming a constant sedimentation rate throughout the Holocene is risky.

Lines 599-602 (Figure 3): These figures do not show “OC concentration” in a), and “OCAR” in b). They only identify the hotspots (>95th quantile) and coldspots (<5th quantile) for both parameters. Please rephrase.

Line 603 (Figure 4): You assume that readers will all know where these seas/gulf/straits/fjords are located on your maps – several do not. You should find a way to show where these water bodies are located either on one of the figures that you show in the main paper, or on an additional figure in the Supplementary Material section.

Line 603, Figure 4: It is obvious that for some of the hotspots, the number/quality of data is not sufficient to provide an acceptable estimation of the transfer efficiencies. This is particularly true for the Beaufort Sea, Chukchi Sea, Gulf of Finland

and W. Scotland and explains why for three of these hotspots, the transfer efficiencies are above 100% (see comment above). Also, the Skagerrak data around 0.2 wt. % is highly suspicious, either because of a measurement/reporting mistake, or because these samples come from a highly different deposition environment. I would not be surprised if the cluster of points at about 0.2 wt.% all come from the same study/group/location. Quality controlling the data would probably have identified these datapoints as problematic.

Line 612, Figure 5: The label here refers to “Mean OC density” in g cm-2, while the same parameter is defined as “OC stock” on line 529 and in the figure caption. The in Figure 5a, the units for the “Total OC stocks” are in Pg only – where is the surface area? I understand that it is a quantity for the entire geomorphic unit, but the same parameter label cannot have different units. Please be consistent.

Line 621, References section: Some journal names are not abbreviated.

Lines 884-888: See my earlier comment on the definition of uncertainty. These two figures only show ranges of values, not uncertainties.

Lines 891-896: Please indicate what “PP” stands for (even though it may seem obvious).

Line 906: “c)” should be “d)”.

Line 923-928 (Table S1): Again, the units are wrong. OCAR units should be “g m-2 yr-1”, and OC density should be “g cm-2”.

Line 942, Table S2: Given their cumulative uncertainties and, in some cases, the very low number of datapoints, the second decimal certainly is not significant for the transfer efficiency percentages. Also, given the number of cores that have been analyzed (n=620, with presumably a large enough proportion of these samples located in the identified hotspots), I don’t understand why only mean values are given rather than mean values and their standard deviations.

Line 991: Are the C/N ratios atomic of mass ratios?

Line 1022 (and Figure S5a): Why showing only the data used to train the model (80% of all the data) and not the entire dataset?

Line 1025, Table S3: Please indicate whether the OC:TN ratios are mass or atomic ratios. Also, in the “Variable name” column, “RESERVOIR” is missing an “R” (twice).

** Visit Nature Portfolio's author and referees' website at www.nature.com/authors for information about policies, services and author benefits**

Communications Earth & Environment is committed to improving transparency in authorship. As part of our efforts in this direction, we are now requesting that all authors identified as ‘corresponding author’ create and link their Open Researcher and Contributor Identifier (ORCID) with their account on the Manuscript Tracking System prior to acceptance. ORCID helps the scientific community achieve unambiguous attribution of all scholarly contributions. You can create and link your ORCID from the home page of the Manuscript Tracking System by clicking on ‘Modify my Springer Nature account’ and following the instructions in the link below. Please also inform all co-authors that they can add their ORCIDs to their accounts and that they must do so prior to acceptance.
https://www.springernature.com/gp/researchers/orcid/orcid-for-nature-research

For more information please visit http://www.springernature.com/orcid

If you experience problems in linking your ORCID, please contact the Platform Support Helpdesk.

Version 2:

Decision Letter:

** Please ensure you delete the link to your author home page in this e-mail if you wish to forward it to your coauthors **

Dear Professor Kamenos,

Your manuscript titled "Hotspots of Arctic and sub-Arctic marine sediment organic carbon are dominated by the Baltic, Barents and Chukchi Seas" has now been seen by our reviewers, whose comments appear below. In light of their advice we are delighted to say that we are happy, in principle, to publish a suitably revised version in Communications Earth &

Environment.

We therefore invite you to revise your paper one last time to address the remaining concerns of our reviewers. At the same time we ask that you edit your manuscript to comply with our format requirements and to maximise the accessibility and therefore the impact of your work.

EDITORIAL REQUESTS:

Please review our specific editorial comments and requests regarding your manuscript in the attached "Editorial Requests Table".

*****Please take care to match our formatting and policy requirements. We will check revised manuscript and return manuscripts that do not comply. Such requests will lead to delays. *****

Please outline your response to each request in the right hand column. Please upload the completed table with your manuscript files as a Related Manuscript file.

If you have any questions or concerns about any of our requests, please do not hesitate to contact me.

SUBMISSION INFORMATION:

In order to accept your paper, we require the files listed at the end of the Editorial Requests Table; the list of required files is also available at https://www.nature.com/documents/commsj-file-checklist.pdf .

OPEN ACCESS:

Communications Earth & Environment is a fully open access journal. Articles are made freely accessible on publication. For further information about article processing charges, open access funding, and advice and support from Nature Portfolio, please visit https://www.nature.com/commsenv/open-access

At acceptance, you will be provided with instructions for completing the open access licence agreement on behalf of all authors. This grants us the necessary permissions to publish your paper. Additionally, you will be asked to declare that all required third party permissions have been obtained, and to provide billing information in order to pay the article-processing charge (APC).

Please use the following link to submit the above items:
Link Redacted
** This url links to your confidential home page and associated information about manuscripts you may have submitted or be reviewing for us. If you wish to forward this email to co-authors, please delete the link to your homepage first **

We hope to hear from you within two weeks; please let us know if you need more time.

Best regards,

Alice Drinkwater, PhD
Associate Editor
Communications Earth & Environment
Consulting Editor
Communications Sustainability

REVIEWERS' COMMENTS:

Reviewer #1 (Remarks to the Author):

Dear Editors and Authors,

I find the manuscript to be substantially improved and I am satisfied with the revisions made. I have no further major concerns. Below are a few final comments, which do not necessarily require another round of revision, but could also be addressed during the potential typesetting or proofreading stage. The line numbers refer to the version of the manuscript without tracked changes:

Line 386: Citations appear without a comma (7374, instead of 73,74)
Line 407: Based on our assessment “of” OC% and 407 OCAR hotspots, we have identified..
Line 640: Please include the link to the data repository for the R-code.

Reviewer #3 (Remarks to the Author):

Thank you for thoroughly and convincingly addressing the comments. I am now satisfied with this revised version and recommend its publication without further modifications. This is a very nice contribution to the field.

** Visit Nature Portfolio's author and referees' website at www.nature.com/authors for information about policies, services and author benefits**

Open Access This Peer Review File is licensed under a Creative Commons Attribution 4.0 International License, which permits use, sharing, adaptation, distribution and reproduction in any medium or format, as long as you give appropriate credit to the original author(s) and the source, provide a link to the Creative Commons license, and indicate if changes were made.
In cases where reviewers are anonymous, credit should be given to 'Anonymous Referee' and the source.
The images or other third party material in this Peer Review File are included in the article's Creative Commons license, unless indicated otherwise in a credit line to the material. If material is not included in the article's Creative Commons license and your intended use is not permitted by statutory regulation or exceeds the permitted use, you will need to obtain permission directly from the copyright holder.
To view a copy of this license, visit https://creativecommons.org/licenses/by/4.0/

REVIEWER #1

General comment

Comment	Response
The study of Langley et al. presents sedimentary OC% and OCAR in the Arctic and sub-Arctic based on an impressive dataset, which is spatially upscaled using 'Quantile Regression Forest (QRF)' modelling. The distributions reveal spatial heterogeneities across the study region, highlighting OC% and OCAR hot- and coldspots, and the authors discuss different potential driving factors for their observations. Furthermore, the spatial distribution is used to quantify carbon stocks and highlight the study region as an important organic carbon sink in a global context. In that, the study provides important and novel results to environmental and climate scientists, especially since the high latitudes are comparably understudied regions and are associated with relatively high uncertainties in previous global model assessments. The methods for data gathering, spatial upscaling and calculations are state-of-the-art, well validated and provide robust results. Regarding relatively high model uncertainties, it is important to consider that this is typical for spatial upscaling, with QRF modelling generally recognised as one of the best performing methods. Figures and tables are well-designed and informative. Nevertheless, I think the manuscript can benefit from major improvements before publication. My main critiques are (1) confusing/wrong terminologies and units, (2) reproducibility of the methods, and (3) interpretation of predictors as key drivers. I address these and additional remarks below. Considering the robust methodology, relevance to the field, and the areas identified for improvement, I would recommend a Major Revision.	Thank you for the thorough and constructive feedback.<br><br>We have revised the manuscript to address the 3 key points raised. Specifically:<br>- We have carefully revised terminologies and units throughout the manuscript.<br>- We have expanded sections of the methods to improve reproducibility, including ensuring that the data and R scripts will be available.<br>- We have revised our interpretation of the predictor variables such that these are now discussed as associations, rather than direct causal drivers of OC%.<br><br>In addition, we have addressed the specific comments below.

Specific comments:

Comment	Response
Line 33: I suggest mentioning QRF as the applied method to spatially upscale the ~14000 datapoints in the abstract.	We have revised the abstract to explicitly state that quantile regression forest (QRF) is the method used for spatial upscaling.
Line 52: Please double-check the bibliography of the first six citations, as they seem to be wrongly attributed in the manuscript.	The citations have been carefully checked, and the bibliography has been updated accordingly.
Line 53-57: I suggest rewriting: "The biological carbon pump, riverine inputs, coastal erosion and glacial discharge deliver OC to the ocean floor, a proportion of which is buried in sediments over millennia". For these broad statements, I suggest citing more general previous work, such as Berner et al. (1980), Early diagenesis: a theoret. approach, and cut the reference to fjords, as they are not a specific focus of this study.	We have revised this sentence following the suggestion here, whilst also incorporating definitions of OC accumulation and burial as per the comment below. The revised version reads:<br><br>*"Marine sediments serve as a vast sink of organic carbon (OC), receiving inputs from the biological carbon pump, riverine sources, coastal erosion and glacial discharge[1,2]. Following deposition at the seafloor, a proportion of OC is retained within sediments as OC accumulation, with a smaller fraction of this accumulated OC subsequently transferred to deeper sediment layers, where it is preserved over millennial timescales (OC burial), representing long-term storage below depths at which OC concentrations remain relatively constant[3,4]."*<br><br>The citations in the opening paragraph have been updated to early works including Berner et al., 1980, Hedges et al., 1997, Burdige, 2007, Hedges & Keil 1995 & Emerson et al., 1988.

Line 64-66: I am not sure if I understand this statement correctly. If we have an understanding of processes contributing to OC accumulation and burial at high latitudes, I assume these processes also contribute to the creation of hot- or coldspots? Consider rewriting. Furthermore, here the terms "accumulation" and "burial" are introduced and I suggest phrasing clear definitions. OC accumulation is the deposition and storage of OC at certain depths (here defined as up to 10 cm sediment depths). OC burial is the long-term storage (calculated at depth below which OC% stays near constant). OC deposition flux/rain rate is the OC flux that reaches the seafloor. I suggest to critically review the usage of these terms throughout the manuscript and adapting them with respect to the definitions set in the introduction.	We have reworded this statement to more clearly reflect our current understanding and better highlight knowledge gaps. This now reads:<br><br>*"While we have a broad understanding of major pathways, sources and processes contributing to marine OC accumulation at high latitudes[10–12], the large-scale spatial distribution of sedimentary OC remains poorly constrained, hindering identification of OC hotspots."*<br><br>We have also provided definitions for OC accumulation and OC burial in the opening paragraph and have reviewed these terms throughout the manuscript to ensure that these match our definitions.
Line 82-83: Consider citing Zhang et al. (2024; https://doi.org/10.1038/s41561-024-01581-4), showing that bottom trawling impact relates to OC contents in the North Sea.	Thank you for the suggestion. We have now cited Zhang et al., 2024.
Line 93-95: Consider citing Diesing et al. (2024) https://doi.org/10.1038/s43247-024-01502-8.	We have now included this citation.
Line 108: What general lengths do the sediment cores have? E.g. "sediment cores (> 50 cm)".	Here we have now stated that we assess the upper 1 m of sediment.
Line 116: spatially	Corrected.
Line 172: "This likely reflects a combination of remineralisation with OC age in deeper sediments". I would assume that deposited OC in the Barents and Chukchi Seas at greater water depths will be more degraded compared to the shallow Baltic Sea. Consequently, I would expect lower degradation rates of the already refractory OC in sediments of the Barents and Chukchi Seas. The argument of lower starting or "initial" OC concentration makes sense and could be the sole argument for explaining why OC concentrations fall below the 1.43 wt% hotspot threshold.	We have revised this explanation to emphasise lower initial OC concentrations as the primary control in explaining why values fall below the 1.43% hotspot value.

Line 185: Please mention how this finding relates to global surface OC storage (i.e. it is approximately 13% (4-29%) of the global 156 Pg number).	Thank you. We have included that our findings represent "*13% (4–29%) of the global marine sedimentary OC stock (155.8 Pg in the upper 10 cm).*"
Line 188-191: For densities, I would expect a unit of g/cm3 instead of g/cm2. Given the magnitude of the numbers, I assume the densities were integrated over the top 10 cm of sediments? If so, please describe that the presented numbers are integrated densities and not volumetric densities.	Thank you for this clarification. The reported values represent depth integrated OC densities over the upper 10cm of sediments (g cm^{-2}) rather than volumetric densities (g cm^{-3}). We have clarified the terminology used in the manuscript accordingly.
Line 191: A suggestion for rephrasing: The elevated OC stock on shelves underscores their importance for high-latitude sedimentary carbon storage. Notably, OC cycling on shelves is highly dynamic given the proximity to land, with changes in terrestrial carbon inputs and marine primary production rapidly influencing deposition patterns, as well as frequent and intense human disturbances to the seabed.	We have rephrased this section following the suggestion.
Line 205: The Arctic has been previously shown as a relatively high OC region, including early work by Berner (1982; https://doi.org/10.2475/ajs.282.4.451) or Hedges and Emerson (1988; https://doi.org/10.1029/PA003i005p00621). Consider citing these early works.	We have included the suggested citations here.
Line 210: I would avoid the term "hotspot" here, as it is defined as being in the top 5% of OC values in this study. A reader could think that the 0.90 wt% Arctic value is in the top 5% of global OC data. I suggest to write "important region in a global context".	We have changed the term 'hotspot' to 'important region in a global context'.
Line 224-226: I find the argumentation about chronology a bit confusing. A temporal analysis (e.g. OC stock generation in the last x years across a region) seems not to be the scope of the sentences in lines 217-224 and of this study in general. I suggest to rephrase the text to "These results suggest that extrapolating surface sediment OC concentrations to deeper sediment layers would introduce large uncertainty in OC stock quantifications.", as	We have removed the chronology argument and rephased as suggested.

this highlights the continuous decrease of OC% with depth below 10 cm at many sites.	
Line 229 - 244: I suggest to restructure this paragraph and add an explanation to the predictive capability of salinity: "High OC% occurs close to land, with the most pronounced hotspots in the semi-enclosed, shallow Baltic Sea (including the Gulf of Finland and Gulf of Bothnia). Here, restricted water exchange with the open ocean and freshwater discharge from surrounding landmasses lead to a pronounced vertical stratification of the water column, creating environmental conditions favourable for OC accumulation35. These stratified conditions likely promote bottom-water hypoxia, suppressing benthic remineralisation and bioturbation and enhancing OC preservation36. Consistent with this, surface and benthic salinity ranked as the highest-importance predictors of OC% (Fig. S2a), with non-linear increases in predicted OC% at lower salinities (Fig. S2b, c). However, it is important to note that salinity itself does likely not have a direct effect on OC% or OCAR, but relates to environmental conditions, such as stratification and hypoxia, that drive OC preservation. Mixed layer depth was another important predictor that is associated with water column stratification, with higher OC concentrations at shallower mixed layers (Fig. S2d). In addition to the stratification effect, a shallow mixed layer can concentrate nutrients and phytoplankton biomass within the euphotic zone, enhancing primary production and the efficiency of OC export to depth37. A further reason for particularly high OC% in the Baltic Sea is a long history of human activities, including sustained nutrient loading, elevating primary production and sedimentary OC inputs35, in contrast to higher-latitude Arctic shelves where anthropogenic influence is weaker and OC% is generally lower." I believe it is important to clarify that there is likely no direct effect of	Thank you for this important clarification. We agree that salinity is unlikely to exert a direct control on OC preservation. Rather, in our QRF approach, salinity therefore more likely acts as a proxy for stratification and associated redox conditions that regulate sediment OC rather than a direct driver. We have revised the text to clarify this point and avoid implying a direct causal relationship between salinity and OC preservation.

salinity on OC preservation. The salinity gradient (not absolute salinity) results in stratification and induces deep water hypoxia, which in turn drives OC preservation. Since the presented predictor is salinity, not salinity gradient (i.e. difference between surface and bottom waters), it may be confusing to the reader to say "salinity drives OC preservation".	
Line 256: The Mackenzie River and the Lena River	Corrected.
Line 259-263: "Consistent with this interpretation of our findings". Consider shortening this to "Consistent with this interpretation". If the authors have data on (long) sediment cores from Arctic coastline areas, it would be interesting to compare the transfer efficiency (Teff) of these areas to the 62% and 77% of the Chukchi and Barents Sea, respectively. If it is old, refractory OC that is deposited along the Arctic coastline hotspots, then it should be reflected as a higher transfer efficiency than the areas with high inputs of fresh marine material, which could strengthen this interpretation.	We have removed 'of our findings'. We agree that a comparison of transfer efficiencies would be valuable in understanding the lability of OC, however data for long sediment cores are limited. Therefore, we do not include this comparison in this paper but agree this is an important direction for future work.
Line 271: Consider citing Zhang et al. (2024); https://doi.org/10.1038/s41561-024-01581-4.	Thank you. Citation included.
Line 275-278: This reads a bit confusing and could need a bit more explanation, especially the "different sediment ages at equivalent depths down core" part in context to OCAR and OC burial being different. OCAR and OC burial can be different from one another, irrespective of age, OC input or sedimentation rates (depending on how they are defined, see line 64). In other words, even if you had a temporal analysis, OCAR and OC burial could still be different.	We have removed the previous implication that this mismatch directly reflects burial efficiency and revised the paragraph to explain that the mismatch between OC% and OCAR primarily reflects variability in sedimentation rate (i.e. dilution effects), rather than OC preservation alone, while noting its indirect influence on preservation via residence time in the surface sediment layer.

Line 282: How does the mismatch in OCAR vs OC% in Fig. 3 translate to OCAR being different from OC burial? OCAR is different from OC burial because of continuous OC remineralisation below 10 cm (which is the cut-off point for OCAR calculations in this study). Please explain or rewrite the sentence.	This paragraph has been reworded following the previous comment.
Line 283: Compiled.	Corrected.
Line 293: The unit of sedimentation rates should be in cm yr-1.	Corrected.
Line 304-305: Consider rewriting this sentence to: Hence, OC may reach marine sediments at an already old and refractory state in river-dominated high Arctic areas, which could partly explain the large range of radiocarbon-derived ages we observed at 1 m sediment depth (Fig. S4).	We have rephased this sentence as suggested.
Line 316: This paragraph seems a bit loose/repetitive to text from above and is not well-connected to the general findings of this study. For example, the study does not show that source-to-sink transits are particularly important in the Arctic compared to other regions globally. Conversely, the study highlights other processes to be important in controlling the distribution of OC% and OCAR, such as the salinity anomaly in the Baltic Sea (and related environmental conditions). Anthropogenic disturbances seem to be another important factor. While source-to-sink factors, such as primary production and riverine inputs, are also mentioned, I am not sure whether this justifies its own paragraph. Especially since the source-to-sink relation and the importance of the highly dynamic shelf areas for OC% and OCAR have been mentioned in other parts of the manuscript. I suggest to merge text compartments of this paragraph with the section 'Bio-environmental controls on organic carbon hotspot creation'.	We agree that the paragraph on source-to-sink carbon pathways was not well integrated with the main findings of the study and partly repeated in earlier text. We have removed this section and incorporated relevant elements into the section 'Bio-environmental controls on organic carbon hotspot creation'. Lines 294-304.

Line 335-338: Regarding the freshening trend of Arctic surface waters and the resulting strengthening of water column stratification in the future: I suggest adding here that contemporary Baltic Sea conditions (enhanced salinity gradient, stratification, hypoxia, nutrient load, etc.) could in part reflect how other Arctic ocean regions may evolve in the future. This also nicely follows up on the idea of Reusch et al. (2018), who highlight the Baltic Sea as ‘a time machine for the future ocean’ (DOI: 10.1126/sciadv.aar8195).	Thank you for this suggestion. We have now highlighted that contemporary Baltic Sea conditions may provide a partial analogue for future Arctic shelf environments under continued freshening, referencing Reusch et al. (2018).
Line 338-354: These text parts read more like a general introduction, as they summarize the effects of warming on the Arctic in general, but they do not directly connect to the main findings of this study. I suggest moving a condensed version of it to the introduction after line 63.	We have revised this section to more explicitly link the described environmental changes to the key predictors identified in our model (e.g. salinity, mixed layer depth, temperature, primary production). The revised paragraphs now directly relate climate-driven changes in the Arctic to the environmental conditions associated with OC hotspots identified in this study.
Line 370-383: “Even though the impact of physical disturbance on sediment OC stocks remains debated (e.g.21–24), using the precautionary principle there is a need to consider whether protection now of Arctic and sub-Arctic carbon stocks or post-damage restoration (if later deemed necessary) is a better option, given other socio-ecological factors.”. I think this text part reads a bit loose and I suggest to merge it with the line above to: “However, such disturbances are projected to increase as a result of diminishing sea ice and a warmer Arctic Ocean25. Given that coastal management at lower latitudes has proven effective in protecting carbon stocks70, there is a need to consider similar strategies for Arctic and sub-Arctic carbon stocks. Based on our assessment of OC% and OCAR hotspots (Fig. 3), we have identified two areas for primary consideration in this context: 1. The high Arctic continental shelf, storing approximately xxx PG of old and young OC that may be vulnerable to future anthropogenic	Thank you for the suggestion. We have tightened this section following your suggestion and those made by reviewer 3.

disturbances. 2. Sub-Arctic hotspots for OC% and OCAR, storing approximately xxx Pg of predominantly young OC, yet ongoing and frequent human disturbances diminish their potential to contribute to atmospheric CO2 removal.”	
Line 385: For the conclusion, I suggest mentioning the total organic carbon stock estimate for the Arctic + sub-Arctic region (19.65 Pg) and how this number relates to global estimates of 156 Pg (i.e. it is an important region because it stores ~ 13% of global surface sedimentary OC). This should also be included in the organic stock section (Line 184) so that it is not mentioned for the first time in the conclusion.	Thank you. The conclusion now includes our predicted Arctic and sub-Arctic OC stock estimates and their relation to global values. We have also incorporated the percentage contribution to global estimates earlier in the manuscript in section ‘The wider Arctic and sub-Arctic marine sedimentary organic carbon stock’. Lines 202-204.
Line 390: “Key controls on this process are salinity, mixed layer depth and benthic primary production explain the variability in OC% across the region, highlighting the complexity of OC burial dynamics.” A word is missing in this sentence (a “which” before “explain”?). Furthermore, as written above, I suggest to formulate interpretations of relationships between predictors and OC contents based on the predictive performance a bit more carefully. While a high predictive performance means that the predictor explains the variability of OC contents well, it does not necessarily establish a causal relationship between predictor and response variable. For instance, salinity is the predictor with the highest predictive performance of the final QRF model results. However, there is (likely) no direct control of salinity on OC accumulation, but rather that salinity gradients indicate the presence of environmental conditions that could be favourable for OC accumulation, which in this study mostly refers to the special conditions in the Baltic Sea (which is a salinity anomaly in the study region) or mass accumulation rates at river mouths (which is not discussed in context to salinity in this study though). This would also better fit with the wider literature, showing correlations and	We have also rephrased this to be more general following your suggestion here and earlier comments.

(partly) direct influence between environmental factors like oxygen, sedimentation rate or grain size with OC% across different regions on the smaller and larger scale. Hence, I suggest to formulate it more generally. For example: “Localised environmental conditions, such as pronounced salinity gradients in the Baltic Sea and elevated particle inputs at river mouths, strongly influence OC% and OCAR and drive the substantial spatial variability across the study region, highlighting the complexity of benthic OC cycling.	
Line 393: For sub-Arctic regions, the discussion has been started already (as cited publications show). Please rephrase this sentence accordingly.	We agree that discussions on the protection of carbon stocks in sub-Arctic regions are already underway and we have revised the sentence to reflect that our findings contribute to, rather than initiate these ongoing discussions.
Line 394: Delete the word “include”?	Corrected
Line 436: I would suggest renaming the paragraph header to “Predictor variables” or “Predictor variables for spatial OC upscaling” since the chosen predictors do not necessarily control OC% or OCAR. They are self-selected (likely limited by what is available in the literature) and have a certain spatial resolution, here 10km. Meaning that finer-scaled features/processes, such as bathymetry (e.g. smaller seafloor depressions), may not be well reflected by the predictor resolution. Further, as written above, a high predictive performance does not always translate to a “control” of the predictor on the response variable.	We have changed the heading to ‘Predictor variables for spatial OC upscaling’ following your suggestion.
Line 450: Is the QRF model based on a previously published R-script? If so, please cite it. If not, I would suggest including the model script for better reproducibility of the results.	The R-script will be made available and in addition we have provided citation where the script was based on previously published model scripts (e.g. model tuning).
Line 451: The more widely used term for this model is “Quantile regression model”. I would suggest using this term throughout the text.	We have corrected this throughout the manuscript.
Line 461-463: Please describe the other applied methods in more detail and how they have been implemented (i.e. several neural	We have revised the text to specify the types of machine learning algorithms evaluated and added references

network and gradient boosting algorithms exist). Alternatively, cite previous applications of QRF models (Diesing et al. (2024), https://doi.org/10.1038/s43247-024-01502-8; Spiegel et al. (2024), 10.3389/fmars.2023.1141448; Friedland et al. (2023), https://doi.org/10.3390/w15183235) to show that QRF is a generally successful method. Friedland et al. (2023) included a comparison between different upscaling techniques in the Baltic Sea, with QRF models showing the best performances.	demonstrating the successful application of QRF in similar environmental and marine sediment mapping studies.
Line 463: I suggest deleting "out-of-the-box".	This has been deleted.
Line 464: I assume it should say high R2 and low RMSE? This would also be consistent with Fig. S7. Otherwise, please revisit the calculations.	Thank you. This has been corrected.
Line 496: I suggest referring to a publication introducing the calculation of permutation-based variable importance.	We refer back to Breiman 2001 here.
Line 506-507: MAR and OCAR units should be g cm-2 yr-1, i.e. grams per square cm per year.	Corrected.
Line 529: OC stock unit should be g cm-2.	Corrected.
Line 542: A pixel or grid cell is 10 km x 10 km or 10 km2.	We have corrected this to 'a resolution of 10 km, corresponding to a pixel size of 10 km x 10 km.'
Line 592: I suggest using "mass accumulation rates" here rather than "sediment accumulation rates" (which could be mistaken with sedimentation rates), as this follows the calculation in Eq. 1 and the cited literature.	We have clarified this to now read 'sediment mass accumulation rates'.
Line 894: Only the top five (not six) predictors are depicted.	The figure caption has been corrected to *'five'* predictors shown.
Line 898: Are the findings of Figure S3 b and c not very similar to the results from the transfer efficiency calculations? If so, one could show the transfer efficiency here rather than introducing a	We agree that the patterns shown in Figure S3b and c are broadly consistent with the transfer efficiency calculations. However, we do feel it is prudent to also remain somewhat conservative here; Fig. S3 is included as a supplementary

second way of calculating the difference between surface and deep sediment OC%.	figure to illustrate the relationship between OC in the upper 10 cm and the upper 1 m, which supports our discussion in section "*The wider Arctic and sub-Arctic marine sedimentary organic carbon stock*." Specifically, it highlights that although our stock estimates are likely conservative (as they are based on the upper 10 cm), simple extrapolation of surface sediment OC to greater depths without accounting for differences between surface and deeper layers would introduce substantial uncertainty. This differs from transfer efficiency, which quantifies the proportion of OC retained over a defined depth interval (to 1 m in this study), rather than explicitly illustrating the relationship between mean 0-10 cm and 0-1 m sediment OC values.<br><br>We have clarified this in the figure caption of Fig S3 which now reads "*…highlighting differences between surface and deeper sediments and potential uncertainty associated with extrapolating surface OC to depth.*"
Line 942: Please explain how a percentage over 100% can be interpreted in the figure description, e.g. OC deposition may have been higher, or OC composition has been different in the past compared to more recently deposited sediments, post-depositional alterations, etc.	Following comments from reviewer 3, we have removed Table 2 due to large uncertainty in transfer efficiency estimates at the sea level, due to limited sediment core coverage. But we are keen to incorporate this suggestion also, so we now clarify in the main text (where transfer efficiencies exceeding 100% are reported for the Kara Sea) that values above 100% may arise from higher OC inputs or differences in OC composition in the past relative to more recent sediments, as well as post-depositional alteration processes.

Reviewer #2

General comments:

Comment	Response
Langlet et "Hotspots of Arctic and sub-Arctic marine organic carbon burial are dominated by the Baltic, Barents and Chukchi Seas" reports on carbon stocks and accumulation rates across the Arctic and sub-arctic seas. I have previously reviews this manuscript and the current form is much improved and an important contribution to ongoing effort to map carbon across our seabed.<br>The surface carbon stock estimates follow a tried and tested approach and produce solid estimates. The interesting portion of the paper is determining the stock to depth (1m) and the accumulation rates.	Thank you for the feedback and we are pleased that you find this version improved.
My only query for the authors is focused on the organic carbon accumulation results. It is unclear how the authors have dealt with non-accumulating areas of the seabed. There are multiple areas in arctic and sub-arctic that do not accumulate or are that are actively being eroded. In other research in the North Atlantic/Arctic an area of applicability approach has been taken to stop the model trying to predict values in non-depositional areas.<br>https://bg.copernicus.org/articles/22/5975/2025/<br>https://iopscience.iop.org/article/10.1088/2515-7620/ae14bc<br>https://www.nature.com/articles/s43247-024-01502-8	In response to your query regarding non-accumulating areas of the seabed, OC accumulation rates (OCAR) were calculated by multiplying mass accumulation rates by OC concentrations. As such, regions that do not accumulate (i.e. mass accumulation rate MAR = 0 g cm^{-2} yr^{-2}) will have a value of 0 g OC cm^{-2} yr^{-2}. As the MAR dataset from Restreppo 2021 does not include negative MARs, our approach does not explicitly capture areas undergoing net erosion. We have clarified this in the methods section under the heading 'OC accumulation rates'. Lines 555-556.
Additionally, there is some discussion of human disturbance, but this does not really come to anything. I would remove this and focus on the mapping elements of the paper.	We acknowledge that this study is primarily based on spatial mapping, however given the relevance of human disturbance particularly bottom trawling, we have decided to keep a brief discussion on this aspect.

For the hotspot analysis it would be good to reference https://iopscience.iop.org/article/10.1088/2515-7620/ae14bc	Thank you for suggesting the hotspots citation. We now reference this paper in the methods section under ‘Hotspots and coldspots identification’.

REVIEWER #3

General comments:

Comment	Response
This an interesting and timely study designed to provide background information on the importance and sensitivity of OC burial hotspots to climate change and anthropogenic activities. It makes use of data available in the literature to try extracting a general picture of OC burial in these critical areas. The study is well designed, and it is presented clearly and efficiently (although with a lot of small mistakes listed in the specific comments, which suggest that the final version of the paper was prepared with haste). It is obvious that the manuscript is the result of an immense amount of work. The writing and visuals are of excellent quality. While this type of study is much needed in these times of climate change and anthropogenic forcing on the organic carbon cycle in marine sediments, I have several concerns with this version of the manuscript as listed in the general and specific comments below.	Thank you for the constructive feedback.
As a first and foremost, the authors should have mentioned in the title and/or the abstract (plus in the list of keywords) that they used machine learning to identify the hotspots and highlight the relative contribution of predictor variables to OC burial. Machine learning is central to this paper and is thus an element that should be rigorously evaluated by someone with the right expertise. I am not an expert in machine learning and therefore I would not have accepted to review this manuscript if I had known that the authors' conclusions were based almost exclusively on machine learning. When contacted by associate editors, potential reviewer have to decide whether they accept to review a manuscript based on the title and abstract only – it is	We agree that the use of machine learning as the methodological approach should be highlighted in the abstract. Accordingly, we have revised the abstract to state the use of quantile regression forests, along with including 'machine learning' and 'quantile regression forest' as key words. We have not incorporated this into the title as it is intended to show the key findings of the manuscript rather than the approach used which is now in the abstract.

thus essential that the abstract reflects the content of the paper, including the methodological approach.	
An important concern with modelling studies is the quality of the input data. Here, the authors have no control on data quality since it was acquired by many different researchers/technicians in lab from all over the world – were all the data included without discrimination? Was an effort made to weed out the apparent bad data? Identifying the obvious outliers (and describing the criteria used to identify them) would allow not only to eliminate the badly measured OC percentages, but also the samples collected in a deposition environment widely different from the majority of the sampling locations in a given area (see below for more on this point).	We agree that data quality is an important consideration when compiling datasets from multiple sources and we now include a 'OC data quality control' section in the methods. Please see the specific comments below regarding quality control on OC data.
Another concern is the use of the word uncertainty to represent the range of possible wt % OC and OCAR values between the 5th and the 95th quantiles. A different qualifier for this range should be used in place of uncertainty since the latter means something very different in statistics. In the Supplementary Material section, the authors use "confidence intervals" for the 5th and 95th limits (not the difference between the two), which is much more accurate than using the word uncertainty as done in this work.	We agree that the term 'uncertainty' may be ambiguous in this context. We have revised the entire manuscript in this regard and now refer to the 5th-95th conditional quantiles, which represent the range of predicted values rather than total uncertainty. Where we have kept the term 'uncertainty' in this context, we now explicitly refer to 'model-based predictive uncertainty'.<br><br>We have also expanded the methods section to clarify what uncertainty sources are and are not presented.
Also, there is no comment/discussion on the precision of their model output aside from providing the R2 and RMSE values in the supplementary material. Yet, non-specialists probably do not know whether the values obtained for the Random Forest model represent a good fit of the modeled values to the measured ones. A quick look at the deviations from the 1:1 line on Figure S7 suggests that the fit is not terribly good – is it? These points should definitely be discussed in the paper to provide the readers with a reasonable idea of the quality of the model outputs.	We have revised the manuscript to provide a clearer assessment of model performance and its interpretation. In the Methods section, we now compare our model performance metrics to previous studies using similar random forest-based approaches. We note that R2 is moderate, which is typical for spatial upscaling of heterogeneous environmental variables such as sedimentary OC.

	Following suggestions from Reviewer 1, we now also reference previous studies demonstrating the suitability of QRF approaches for spatial prediction of OC compared to other machine-learning approaches.
Linked to the point above, the authors should absolutely explain why they have not taken into account the uncertainties associated with the sediment accumulation rates (SAR) taken from Restreppo et al. in their model development. This is particularly important since Restreppo et al. specifically mention that their "estimates for the mass of glaciated/ice rafted sediments exported to the oceans are not precise", a statement that should raise a red flag when their modelled data is used in (sub-)Arctic environments.	We acknowledge that the prediction quantiles presented for OCAR currently reflect only uncertainty arising from the model structure and from observed variability in OC in the training data for similar predictor conditions and do not incorporate uncertainty in MAR from Restreppo et al. While Restreppo et al. note that uncertainties may be substantial in some environments, these uncertainties are not available in a form that could be directly propagated with our OC% conditional quantiles. We have clarified this in the manuscript and now discuss in the section 'Organic carbon accumulation rate hotspots' how regions of higher MAR uncertainty may translate to greater uncertainty in our OCAR estimates.
While the list of predictor variables may look complete to the non-specialist, it is missing a critical control of OC preservation in marine sediments: the effect of minerals on the wt.% values. One the one hand, mineral matter dilutes to varying extents the organic content in a sample, which influences the resulting wt.% OC values. Because mineral dilution is somewhat taken into account through the use of SAR, OCAR is a much more solid proxy for the identification of hot or cold spots compared to wt.% OC. On the other hand, minerals provide physical protection against OC remineralization in the sediment. Iron hydroxides are particularly critical in that respect: through direct interaction with iron hydroxides, between 10-50% of total sedimentary OC (rough numbers) has been shown to be protected from mineralization. It would have been easy to add reactive iron as a predictor variable in the input data since more and more studies report this value. Clays are also providing protection	We agree that mineral interactions play a key role in OC preservation, both through dilution effects and through mineral protection mechanisms (e.g., association with iron hydroxides and clay minerals). While reactive iron and clay content would be valuable predictor variables, these data are not currently available at sufficient spatial coverage to be included in a pan-regional modelling framework such as ours. As noted by the reviewer, mineral influence is indirectly captured through predictor variables included in our study (e.g., sedimentation rates and environmental proxies), although we recognise that this does not fully resolve the role of mineral protection mechanisms. We have revised the methods section 'Predictor variables for spatial OC upscaling' to acknowledge the role of mineral-associated OC preservation, including iron oxides and clay minerals, and highlight this as an important avenue for

against OC remineralization, although I suspect that clay content is not a variable that is often reported in the literature (not to mention that not all types of clays are equal in this respect).<br><br>Because of these concerns (above and below), this paper should be accepted with major modifications only is a machine learning specialist evaluates favorably this contribution.	future work as more spatially resolved datasets become available.

Specific comments:

Comment	Response
Line 116: “spatial” should be “spatially”.	Thank you. Corrected.
Lines 119-120: Please see comment below (comment referring to lines 492-493) regarding the definition for uncertainty used here. What is labelled “uncertainty” in this work is a range of possible values for the samples that are not tagged as coming from a hotspot (>95th quantile) or a coldspot (<5th quantile). I agree with the way this range is presented in the next paragraph (5th-95th conditional quantiles) as this terminology much better represents what these values mean.	We have revised the text to refer to the $5^{th-}95^{th}$ conditional quantiles, which represent the range of predicted values rather than total uncertainty. We clarify that the uncertainty presented is the model-based predictive uncertainty.
Line 125 and elsewhere in the text: The double parentheses are confusing. Rather than inserting the ranges between the medians and the units, I suggest listing them separately since word count is not a critical issue for Comms. Earth & Environ.	The median and ranges have now been listed separately throughout the text.
Lines 121-141: None of these locations are indicated on the maps. Not every reader is an expert in Geography or Oceanography. Please find a way to indicate them on the maps (or on an additional figure in the Supplementary Material section).	Thank you for this suggestion. We have now included sea delineations and labels in Figures 1 & 2.
Lines 130-131: Please add the “wt.%” units next to the listed values.	Thank you. We have included the units (wt.%) where they were accidently missed here.

Lines 144-160: The units for OCAR are “g m-2 yr-1”, not “g m2 yr-1”. Please correct everywhere.	We have corrected these units and thoroughly check the rest of the manuscript to ensure the correct unites are reported.
Lines 145-146: Remove one of the “and” in the parentheses.	The terminology is defined by the IHO. We have adjusted from ‘North Pacific Ocean and Southeast Alaska and British Columbia’ to ‘North Pacific Ocean and Coastal Waters of Southeast Alaska and British Columbia’.
Lines 162-182: None of the calculated efficiencies higher than 100% are discussed here, as they should be. A preservation efficiency higher than 100% either indicate a major change in the OCAR with time (an increase), unreliable data (which brings back the issue of data selection and quality control on the input data used in the model), samples coming from widely varying deposition centres (which is also a problem), or a widely insufficient number of datapoints (which again is a serious problem). These points should definitely be addressed.	We now clarify that values above 100% (e.g. Kara Sea) may arise from higher OC inputs or differences in OC composition in the past relative to more recent sediments, as well as post-depositional alteration processes.<br><br>Please also see response below regarding Table 2.
Also, lines 162-182: Please see comment on Table S2 (line 942) regarding the uncertainties on transfer efficiencies.	See response below regarding Table 2
Lines 187-188 and Table S1: The percent contributions should not be listed with two decimal points – this is misleading considering the cumulative uncertainties associated with them.	We have removed the decimal points for reported transfer efficiencies.
Lines 190-191 and 216: The units should be “g cm-2”, not “g cm2”.	This has been corrected and checked elsewhere in the manuscript.
Line 230: Replace the second “including” by “and”.	Corrected.
Lines 237-238: Not necessarily hypoxia, which is defined with a clear dissolved oxygen concentration threshold. The key factors here are oxygen penetration depth (OPD) and exposure time (OET) in the surface sediment. Simply decreasing dissolved oxygen level concentrations, even when above the defined threshold for hypoxia, has a negative influence on OPD and OET,	We have revised the text to avoid implying hypoxia and instead reflect the role of OPD and OET in controlling OC preservation. The revised sentence now reads:<br><br>*“These low-salinity, stratified conditions likely reduce oxygen availability at the sediment-water interface, decreasing oxygen penetration depth and oxygen exposure time, in turn,*

thus partly reducing the extent of OC remineralization. Please rephrase.	*suppressing benthic remineralisation and bioturbation and enhancing OC preservation[40]."*
Lines 266-271: This spatial heterogeneity of deposition environments is indeed a major limitation of this work. The sample set used in this work might not reflect the diversity and relative proportion of the different types of deposition environments, which might introduce unquantifiable biases in the model results. While it is impossible to identify and quantify such biases with the available dataset, a warning to this effect should at least be added somewhere within the paper (not in the Supplementary Material). The best place would be just after line 271.	We agree that spatial heterogeneity in depositional environments may not be fully captured by the available sample set and could introduce unquantified biases. We have added a statement in the main text (after line 271) to acknowledge this limitation.
Lines 280-282: This mismatch could also be due to mineral dilution, which have not been taken into account in this work. The most important parameter to identify hotspots is OCSA, not %OC as OCAR is independent of dilution by minerals. This is also why neglecting to consider the uncertainty on the SAR data used in this work (see comment below) is in my opinion a major limitation.	We have revised this section which now discusses mineral dilution as a factor contributing to the mismatch between hotspots of OC% and OCAR.<br><br>We acknowledge that uncertainty in SAR/MAR is an important limitation. However, these uncertainties are not available in a form that can be directly propagated within our QRF approach (not fully quantified). Thus, when reporting OCARs in the section 'Organic carbon accumulation rate hotspots', we have highlighted that OCAR uncertainty reflects only the model-based predictive uncertainty in OC and does not include SAR/MAR uncertainty, and we now discuss how this leads to higher OCAR uncertainty in regions with high MAR uncertainty.
Line 283: "Complied" should be "Compiled".	Corrected
Line 293: Why are most of the units wrong?!? Here, "cm2 yr-1" should be "g cm-2 yr-1". These repeated mistakes cast doubt on the calculations performed in the model or in the care taken when writing the paper. Also, do you mean 4x10-5 to 10.9x10-5? If yes, then why is there only one sig fig for the lower number of the range and three for the higher one?	Thank you for highlighting these errors. The units have corrected the units to cm yr^{-1} as these values refer to sedimentation rates rather than mass accumulation rates. We have also revised the reported ranges to improve readability.

Line 295: The separator for the thousands should be a coma, not a space ("26 000" should be "26,000").	Thank you. Corrected.
Lines 296-297: Not necessarily. In the Arctic, the reservoir effect (time spent on land before being delivered to and buried in margin sediments) can be very high. This reservoir effect is not accounted for in the way this sentence is written ("buried" should be removed since the age reflects both the reservoir effect and the time the OC was buried in sediments).	We agree with this point. The word buried has been removed from this sentence.
Lines 311-312: This sentence is not clear as written. The word "modelled" should be added before "time available" on line 311 ("…affects the modelled time available…").	We agree that the original sentence was unclear. As our study models OC age from radiocarbon data rather than remineralisation timescales, we have revised the sentence to clarify that OC age reflects burial history, sediment mixing and the incorporation of pre-aged material, and does not directly represent the time available for remineralisation since deposition.
Lines 316-325: In fact this change (line 324) is already occurring, which means that the assumption of steady-state SAR and OCSA is shaky at best. These recent changes have most probably affected the model outputs. Given that we have a very good idea of the direction of these changes (as discussed in the following section), the effect of such unaccounted changes on the current model outputs should be mentioned and discussed in this section. The next section only discusses the future potential effects of climate change.	We have revised the paragraph (which is now integrated in the section 'Bio-environmental controls following comments from Reviewer 1) to reflect that changes in terrestrial OC fluxes are already occurring and to acknowledge that model outputs may have higher uncertainties in these rapidly changing regions.
Line 338: The scientific meaning of "Atlantification" should be given for the non-specialist readers.	We have included a definition of this term.

Lines 358-359: This is a highly debatable conclusion. While it might not help reducing “recent” CO2 emissions, the burial of old permafrost carbon in high Arctic river shelves help reduce current and near future emissions of CO2. Old OC age might not always mean highly refractory OC. Large pools of OC can simply be trapped and shielded from degradation in a frozen compartment, thus aging in situ without its usual concurrent “refractorization”.	We agree that burial of old carbon can still play a significant role in limiting present and future CO_2 emissions. We have revised the text to reflect this.
Lines 411-435: What was the data quality control framework followed to determine good vs. bad data? Were all of the data included without identifying potential outliers? As you know, the quality of the data available from the literature can be highly variable. Please provide information on this point.	We agree that data quality is an important consideration when compiling datasets from multiple sources. We also acknowledge that the dataset is derived from multiple datasets/sources, involving different sampling and analytical approaches, which introduces inherent uncertainty. All data were screened for obvious inconsistencies and implausible values by assessing the data distributions (now shown as histograms in the revised manuscript, Fig. S5) and where possible, cross-checked against associated metadata. However, no data points were excluded solely on the basis of being statistical outliers as these values may reflect genuine environmental variability rather than measurement error, particularly given the wide range of depositional environments represented in the dataset. We now include a ‘OC data quality control’ section in the methods. Lines 458-465.
Lines 444-448: Given that the number of datapoints for some of the predictor variables was probably much lower than for other more routinely available, did you evaluate the quality of the 10 km grid reprojections for each predictor? How? Can you provide an assessment of the effect of native data density on the quality of these reprojection, and thus on the modelled influence of these predictor on the identification of OC wt% and OCSA hot/cold spots?	The majority of predictor variables were obtained from Bio-ORACLE, which provides globally gridded environmental layers that have already been standardised to a common spatial resolution (5 arc-min) through interpolation and gap-filling of underlying observational and modelled datasets.<br><br>All predictors were reprojected to an Arctic projection and resampled to a 10 km grid, which was selected to match the coarsest input resolution and avoid introducing artificial

	spatial detail. The resampling therefore represents a harmonisation step rather than interpolation of sparse raw data. This is clarified on lines 478-481.
Lines 492-493: While the categorisation of the hotspots (>95th quantile) and coldspots (<5th quantile) makes sense, the definition used in this work for the “uncertainty” as the difference between these two limits does not. What you are defining here is a range of possible values for samples that are neither hot or cold spots. The word uncertainty means something very specific in statistics, and your definition on these two lines does not correspond to the universally accepted meaning of this word. This point raises the issue of uncertainty in the identification of the hotspots and coldspots, which is nowhere to be found in this paper.	Please see previous comments regarding the term uncertainty.<br><br>We have now also incorporated prediction uncertainty in identification of hotspots and coldspots, using the 5th and 95th conditional quantiles from the QRF to show regions of high and low certainty. These changes are described in the methods and shown in Figure 3.
Line 506-507 and 511: OCAR units should be “g cm-2 yr-1”, not “g-1 cm2 yr-1”, as they are indicated on Figure 2.	Thank you for highlighting this mistake. The units have been corrected.
Line 510-511: Why was the uncertainty of the sediment mass accumulation rates of Restreppo et al. not used? Their uncertainties have a significant influence on the quality of the output data from your model given the fact that Restreppo et al. specifically stated the following: “Additionally, the estimates for the mass of glaciated/ice rafted sediments exported to the oceans are not precise”. A reason for not considering this additional source of uncertainty should be provided in the text, and the uncertainties in sediment MAR as calculated in Restreppo et al. should be discussed. It is important to know whether the hotspots calculated here, without taking into account the uncertainties in MAR, correspond to areas of high (or low) MAR uncertainties. These details are important given the approach used to define the hotspots and coldspots in the following paragraph.	Please see above comments regarding uncertainty on sediment MARs.

Line 529: The units for OC stock should be “g cm-2” rather than “g-1 cm2”.	Corrected.
Lines 533-534: Again, this is not an “uncertainty” as understood by most.	Thank you. This has been revised to prediction intervals.
Line 552: Is this a safe assumption (constant sedimentation rate)? Given that the Arctic is the area most affected by global warming, one would expect significant changes in the recent sedimentation rates. Please comment. The assumption of constant bulk density is safer but given the magnitude of the changes affecting the (sub-)Arctic environment, assuming a constant sedimentation rate throughout the Holocene is risky.	We have removed the statement regarding the assumption of constant sedimentation rates, as transfer efficiency was calculated as a function of depth rather than time. As such, sedimentation rates were not explicitly incorporated into the calculation.
Lines 599-602 (Figure 3): These figures do not show “OC concentration” in a), and “OCAR” in b). They only identify the hotspots (>95th quantile) and coldspots (<5th quantile) for both parameters. Please rephrase.	We have revised the figure caption to make clear that panels a and b show hotspot and coldspot regions, rather than continuous OC concentration and OCAR values.
Line 603 (Figure 4): You assume that readers will all know where these seas/gulf/straits/fjords are located on your maps – several do not. You should find a way to show where these water bodies are located either on one of the figures that you show in the main paper, or on an additional figure in the Supplementary Material section.	Thank you for this suggestion. We have included sea delineations and labels in Figures 1 & 2. These figures also now show the location of major Arctic rivers that are discussed in the main text.
Line 603, Figure 4: It is obvious that for some of the hotspots, the number/quality of data is not sufficient to provide an acceptable estimation of the transfer efficiencies. This is particularly true for the Beaufort Sea, Chukchi Sea, Gulf of Finland and W. Scotland and explains why for three of these hotspots, the transfer efficiencies are above 100% (see comment above). Also, the Skagerrak data around 0.2 wt. % is highly suspicious, either because of a measurement/reporting mistake, or because these samples come from a highly different deposition environment. I	Thank you for this important point and we agree that transfer efficiencies in some seas are poorly constrained due to limited core coverage. In response, we have revised our analysis with additional quality controls and transparency as follows:<br>i) Restricted calculation to cores with data in both the 0 – 10 cm and 90 – 100 cm depth interval.

would not be surprised if the cluster of points at about 0.2 wt.% all come from the same study/group/location. Quality controlling the data would probably have identified these datapoints as problematic.	ii) Estimated transfer efficiencies at a both pan Arctic & sub-Arctic and regional scale (e.g. Baltic region) rather than for individual seas<br>iii) Included error bars that represent the standard deviation of mean OC% for each depth interval.<br>iv) Added a map to Figure 4 of the distribution of sediment cores to show coverage across hotspot regions<br>v) Included histograms alongside the OC profiles to show the number of OC data points and number of cores used to calculate mean OC%.<br>vi) Calculated and reported standard deviations for transfer efficiencies.<br><br>We hope that these revisions avoid overinterpretation of poorly constrained estimates and better reflect the uncertainty associated. We have also revised our discussion under 'Organic carbon concentration hotspots in sub-surface sediments' accordingly. Lines 175-198.
Line 612, Figure 5: The label here refers to "Mean OC density" in g cm-2, while the same parameter is defined as "OC stock" on line 529 and in the figure caption. The in Figure 5a, the units for the "Total OC stocks" are in Pg only – where is the surface area? I understand that it is a quantity for the entire geomorphic unit, but the same parameter label cannot have different units. Please be consistent.	Thank you for highlighting the inconsistency. We have revised these terms in Figure 5 and throughout the manuscript, distinguishing between depth-integrated OC density (g cm^{-2}) defined over the upper 10 cm of sediment and OC stock (Pg) which is calculated by multiplying depth-integrated OC density by the area of seas or geomorphic units.
Line 621, References section: Some journal names are not abbreviated.	The reference list has been checked, and journal names have been abbreviated.
Lines 884-888: See my earlier comment on the definition of uncertainty. These two figures only show ranges of values, not uncertainties.	Thank you. Revised.

Lines 891-896: Please indicate what “PP” stands for (even though it may seem obvious).	We have written this is full in the figure (‘Benthic primary production’).
Line 906: “c)” should be “d)”.	Thank you. Corrected.
Line 923-928 (Table S1): Again, the units are wrong. OCAR units should be “g m-2 yr-1”, and OC density should be “g cm-2”.	Thank you for highlighting this mistake. This has been corrected.
Line 942, Table S2: Given their cumulative uncertainties and, in some cases, the very low number of datapoints, the second decimal certainly is not significant for the transfer efficiency percentages. Also, given the number of cores that have been analyzed (n=620, with presumably a large enough proportion of these samples located in the identified hotspots), I don’t understand why only mean values are given rather than mean values and their standard deviations.	As per previous comments, Table 2 has now been removed as transfer efficiencies were not calculated for individual seas. Were regional transfer efficiencies are reported within the main text, these are reported with standard deviations and to 1 decimal place.
Line 991: Are the C/N ratios atomic of mass ratios?	OC:TN ratios were compiled from multiple sources, which did not consistently specify whether values were reported on a mass or atomic basis. We have noted this in the supplementary material. However, as these ratios are not used in the analyses in this manuscript, this uncertainty does not affect the results we present.
Line 1022 (and Figure S5a): Why showing only the data used to train the model (80% of all the data) and not the entire dataset?	Thank you for highlight this. This was worded incorrectly and it is in fact the entire dataset. We have corrected.
Line 1025, Table S3: Please indicate whether the OC:TN ratios are mass or atomic ratios. Also, in the “Variable name” column, “RESERVOIR” is missing an “R” (twice).	We have noted that OC:TN are reported as mass or atomic ratios in the ‘Description’ column in Table S3.

Dear Editor,

Please find our response to reviewer comments below.

Reviewer 1

Comment	Response
Line 386: Citations appear without a comma (7374, instead of 73,74)	On re-reading, we realised these two references were placed wrongly and not relevant to the sentence, we have now removed them
Line 407: Based on our assessment "of" OC% and 407 OCAR hotspots, we have identified..	The word "of" has been added as requested.
Line 640: Please include the link to the data repository for the R-code.	The figshare link including the R code is now in the Data Availability statement. The doi is 0.6084/m9.figshare.31126942

Reviewer #3 (Remarks to the Author):

Comment	Response
Thank you for thoroughly and convincingly addressing the comments. I am now satisfied with this revised version and recommend its publication without further modifications. This is a very nice contribution to the field.	No response required
